# A Comparative Review on Applications of Different Sensors for Sign Language Recognition

**DOI:** 10.3390/jimaging8040098

**Published:** 2022-04-02

**Authors:** Muhammad Saad Amin, Syed Tahir Hussain Rizvi, Md. Murad Hossain

**Affiliations:** 1 Department of Computer Science, University of Turin, 10149 Turin, Italy; 2 Department of Electronics and Telecommunication (DET), Politecnico di Torino, 10129 Torino, Italy; 3 Department of Modelling and Data Science, University of Turin, 10149 Turin, Italy; md.hossain50@edu.unito.it

**Keywords:** sign language, machine learning, artificial intelligence, supervised and unsupervised learning, sensor, gesture recognition, man-machine interface, flex sensors, gyroscope, accelerometer sensor, support vector machine, K-Nearest Neighbor, discriminant analysis, decision tree

## Abstract

Sign language recognition is challenging due to the lack of communication between normal and affected people. Many social and physiological impacts are created due to speaking or hearing disability. A lot of different dimensional techniques have been proposed previously to overcome this gap. A sensor-based smart glove for sign language recognition (SLR) proved helpful to generate data based on various hand movements related to specific signs. A detailed comparative review of all types of available techniques and sensors used for sign language recognition was presented in this article. The focus of this paper was to explore emerging trends and strategies for sign language recognition and to point out deficiencies in existing systems. This paper will act as a guide for other researchers to understand all materials and techniques like flex resistive sensor-based, vision sensor-based, or hybrid system-based technologies used for sign language until now.

## 1. Introduction

A speaking or hearing disability is a cause by which people are affected naturally or accidentally. In the world’s whole population, there are approximately 72 million deaf-mute people. Lack of communication is seen between ordinary and deaf-mute people. This communication gap affects their whole lives. A unique language based on hand gestures and facial expressions lets these affected people interact with the environment and society. This language is known as sign language. Sign language varies according to the region and native languages. However, when we speak of standards, American Sign Language is considered as a standard for number and alphabets recognition. This standard is considered as the best communication tool for affected people only. An average healthy person with all abilities to speak and hear is not required to know this prototype because that person is entirely unfamiliar with these signs. There are two ways to make communication feasible between a healthy and affected person. Firstly, convince that healthy person to learn all sign language gestures for communication with the deaf-mute person or, secondly, make any deaf-mute person capable of translating gestures into some normal speaking format so everyone can understand sign language easily. Considering the first option, it almost looks impossible to convince any healthy person to learn sign language for communication. This is also the main drawback of sign language. Therefore, technologists and researchers have focused on the second option to make deaf-mute people capable of converting their gestures into some meaningful voice or texture information. For Sign language recognition, a smart glove embedded with sensors was introduced that can convert handmade gestures into meaningful information easily understandable by ordinary people.

Smart technology-based sign language interpreters that remove the communication gap between normal and affected people use different techniques. These techniques are based on image processing or vision-sensor based techniques, and sensor fusion-based smart data glove-related techniques, or hybrid techniques. No such limitations are seen in these technological interpreters as extracting required features from an image usually creates problem due to foreground and background environmental conditions. If we consider an image or vision-sensor based recognition system, there is no limitation of foreground or background in gesture recognition. Considering a sensor-based smart data glove, there is no limitation of carrying this data glove as it is mobile, lightweight, and flexible. Research has shown that many applications based on vision-sensors, flex-based sensors, or hybrid techniques with different combinations of sensors are currently being used for a communication tool. These applications also act as a learning tool for normal people to comfortably communicate with deaf or mute people. Latest technologies like robotics, virtual reality, visual gaming, intelligent computer interfaces, and health-monitoring components use sign language-based applications. The goal of this sign language recognition-based article was to deeply understand the current happenings and emerging techniques in sign language recognition systems. This article completely reflected on the evolution of gesture recognition-based systems and their performance, keeping in mind the limitations and pros and cons of each module. The aim of this study was to understand technological gaps and provide analysis to researchers so they can work on highlighted limitations in future perspectives. So, the aims and objectives of the prescribed study were fulfilled by considering published articles based on the specified domain, the technology used, gestures and hand movements recognized, and sensor types and languages targeted for recognition purposes. This paper also reflected on the method of performance evaluation and effectiveness level achieved for analyzing sign language techniques used previously.

For sign language recognition, fingers bend and hand position is explicitly considered. Gesture location and movement of the body and hands collectively perform any sign translation. For recognizing and identifying any sign made for some specific language, recognition factors play an important role in this scenario. These recognition factors include facial expressions, hand orientation, head and body movement types, finger configuration, and articulation point analysis as shown in Table 1. Any sign made by the deaf-mute person is a combination of these factors. Automated intelligent systems use these factors for the recognition of gestures.

People with speech and hearing disabilities use sign language based on hand gestures. Communication is performed with specific finger motions to represent the language. A smart glove is designed to create a communication link of individuals with speech disorders. It provides a close analysis of the engineering and scientific aspects of the system. The fundamentals are taken into account for the social inclusion of such individuals. A smart glove is an electronic device that translates sign language into text. This system is designed to make communication feasible between the mute people and the public. Sign language recognition-based techniques consist of three main streams that are vision-sensor based, flex sensor-based, and a combination of both vision sensor and flex sensor fused systems and are listed below.

1. Vision-sensor based SLR system
2. Sensor-based SLR system
3. Hybrid SLR system

**Figure 1:**
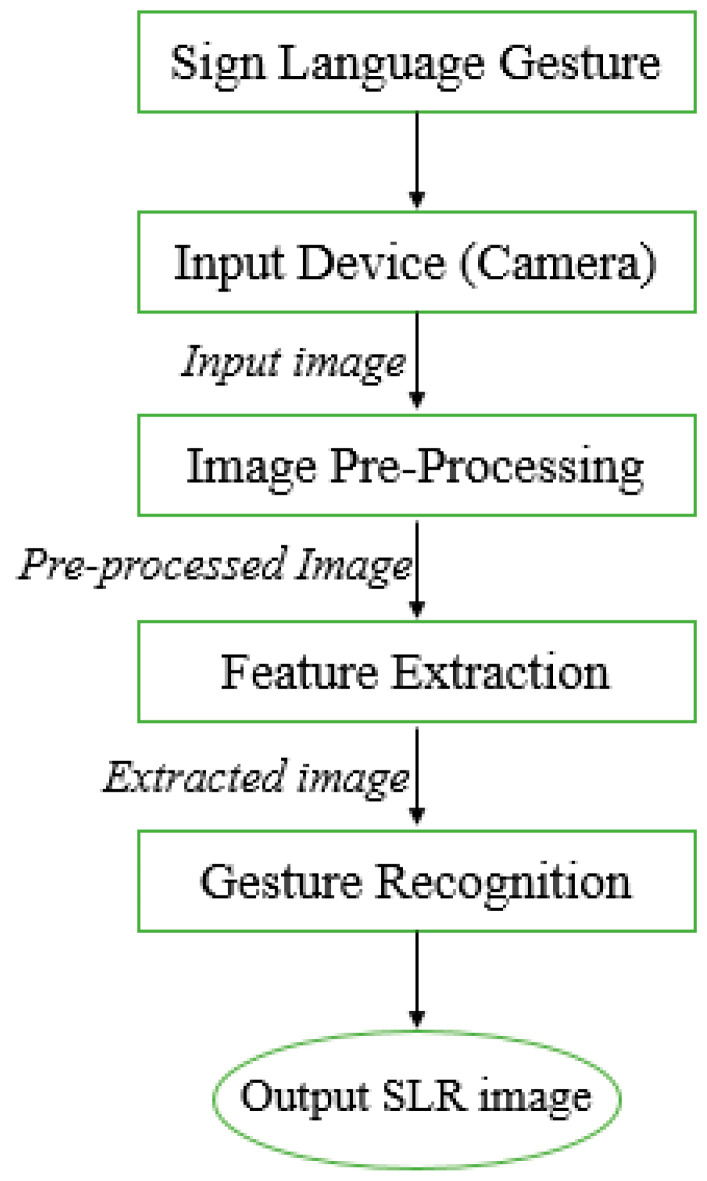
Processing steps for the vision sensor-based SLR system.

In vision-sensor based systems, input data are captured using a high-definition camera, mostly Kinect cameras, for input-processing purposes. This captured input image is processed and recognized by matching it with sign language images placed in the database. The main advantage of using the vision-sensor based method is relief from sensors. The sensor-based model is costly compared with the vision-based model, which requires only an HD camera. The laptop’s built-in cameras are avoided as they produce blur images for processing. In the case of the blurred input image, the pre-processing cost is increased. Despite the advantages, there are some disadvantages of using a vision-sensor based approach for sign recognition. In a camera-based model, a field of view is limited for capturing input data. The number of cameras increased as we dealt with occlusion and depth-related issues in image-based models. The increased number of cameras also increased the computational cost of the overall system. A systematic model of steps involved in the vision-sensor-based sign language recognition system is shown in Figure 1.

**Figure 2:**
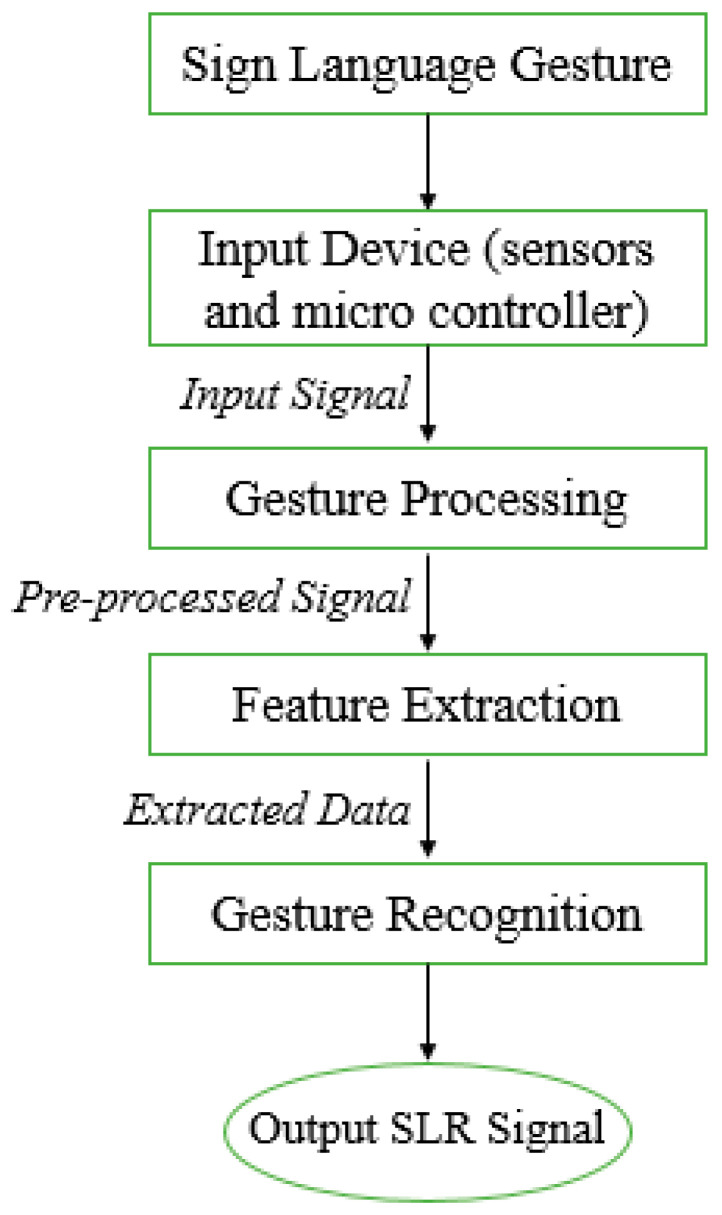
Processing steps for the sensor-based SLR system.

A smart glove embedded with different sensors is used for gesture recognition in the sensor-based model. These sensors include flex sensors for measuring figure bending, accelerometers for angle and degree movement, and abduction and proximity sensors. These sensors are attached to the user’s fingers and on the upper side of the palm; therefore, the length of the sensor can vary according to finger length. The sensors provide outputs in analog form. Value variation is determined by the degree of finger bending. The value provided by the sensor will be given to the controller. It processes the signals and performs analog to digital signal conversion. At this stage, the data are in the form of digits having some specified values according to the sign made for language. These sign values are collected and stored in a file to perform actions. The major advantage of using different sensors is the accomplishment of direct values related to some specific sign made according to some alphabet or digit. So, there is no need for pre-processing as in the vision-sensor-based model. A systematic model of steps involved in a sensor-based sign language recognition system is shown in Figure 2.

**Figure 3:**
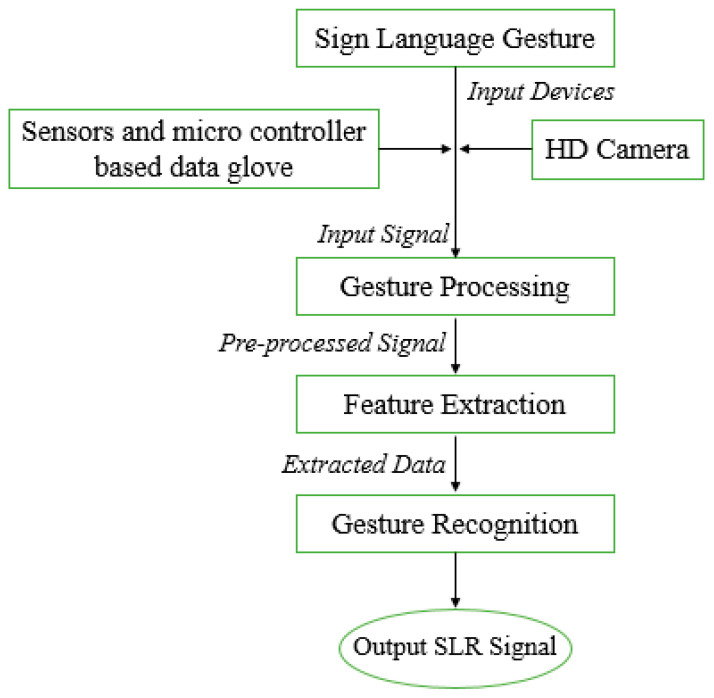
Processing steps for the hybrid SLR system.

A hybrid system for sign language is a combination of both vision-sensor-based and combination of different sensors based models. So, glove and camera-based orientation are collectively used for raw gesture data collection. Data accuracy and precision are determined using the mutual error elimination method. This method removes errors for data accuracy. Due to complex and large computations and cost overhead, this method is not commonly used for data collection. However, hybrid methods of data collection are used in augmented reality and virtual reality-based systems, which somehow produce promising results related to sign language gesture-based data collection. Modern techniques are moving towards advanced sensors and vision-sensor-based sign recognition approaches. Processing steps for the hybrid recognition model are shown in Figure 3 given below.

A comprehensive study related to sign language recognition was also conducted. Review-based articles played a vital role in understanding sign language fundamentals. The general review paper reflected application-based discussion with several pros and cons. Emerging trends, developing technologies, and sign-detection device characteristics were the focus of general review articles. Sign language recognition-based review articles covered most techniques achieved for gesture recognition. The focus of these articles was on technology. Choice of the right sensory material and limitations in existing systems are reflected in these articles. So, these articles provide a deep understanding of recognition materials and methods used to obtain an efficient sign dataset with maximum accuracy. A detailed analysis of sensor based, vision based, framework based, commercial, non-commercial and hybrid systems covering the conceptualization of review articles will be discusses in Section 2 of this paper. Machine learning is an emerging technique with very high efficiency and good output response. Mainly machine learning is used for training systems that will perform actions intelligently. Basically, any machine can be made intelligent with the help of the machine learning approach. Machine learning techniques are counted as under the tree of artificial intelligence. So, the data by which our algorithm learns to perform operations were provided by the sensor data collected in a file. These data were used for training purposes. In this way, the gesture is recognized, and the algorithm efficiency can be computed via testing operations. With the help of this technique, the barrier faced by mute people in communicating with society can be reduced to a great extent when someone wants to share with people who are not able to speak due to a vocally impaired disability. This communication gap creates a problem. Therefore, sign language is used for communication. There are several people who cannot understand these sign gestures. Communication hindrance between the public and mute people is the main problem to be addressed.

## 2. Literature-Based Sign Language Recognition Models

A lot of image processing and sensor-based techniques have been applied for sign language recognition. Recent studies have shown the latest framework for sign recognition with the advent of time. Detailed literature analysis and a deep understanding of sign language recognition categorized this process into different sub-sections. The further division was completely application-based depending upon the type of sensors used in the formation of data gloves. So, these subdivisions were based on non-commercial prototypes for data acquisition, commercial data acquisition prototypes, Bi channel-based systems, and hybrid systems. Introducing non-commercial systems, these prototypes are self-made systems that use sensors to gather data. These sensor values are transmitted to another device, usually any processor or controller, to understand transmitted sensor data and convert these data into their respective sign language format. Most of the sensor-based systems are non-commercial prototype-based systems discussed in the literature review. In non-commercial systems, most of the authors worked on finger bend detection regarding any sign made. So, a large variety of different solo sensors or combinations of different sensors were used to detect this finger bending. So, SLR models can be further divided into non-commercial prototype-based and framework-based prototypes. Different literature review-based models are discussed in detail in the sections below.

### 2.1. Sensor-Based Models

An American Sign Language Recognition system with flex and motion sensors was implemented in ref. [1]. This system produced better results than a cyber glove embedded with a Kinect camera. Authors succeeded in proposing a model which performs better recognition of signs using different algorithms of machine learning [2]. Response time and accuracy were increased using better sensors and an efficient algorithm for the specified task. The traditional approach of sign language was changed by embedding sensor networks for recognition purposes. A proposed model was implemented by the combination of Artificial Neural Network (ANN) and Support Vector Machine (SVM) in sign language recognition. This combined algorithm produced better results than the Hidden Markova model (HMM) [3]. A smart data glove named “E-Voice” was developed by authors for alphabetical gesture recognition of ASL. The prototype was designed using flex sensors and accelerometer sensors. The data glove was successful in recognizing sign gestures with improved accuracy and increased efficiency [4]. Sign language is a subjective matter, so a new method of recognition was developed using surface electromyography (sEMG). Here, sensors were connected to the right forearm of the subject and collected data for training and testing purposes. They used the Support Vector Machine (SVM) algorithm for recognition and obtained better results for real-time gesture recognition [5]. Another model was proposed as a combination of three types of sensors. These sensors included flux, motion, and pressure sensors to determine SVM impact on sign language recognition [6]. Daily activity was recognized using a smart-data glove. Two basic techniques for gesture interpretation were used using data glove interaction [7].

Implementation of a more advanced approach including a deep learning model was developed in ref. [8]. Static gestures were converted into American Sign Language alphabets using Hough Transform, and this technique was applied on 15 samples per alphabet and obtained 92% accuracy. A combination of motion tracker and the smart sensor was used in sign language recognition. An Artificial Neural Network approach was implemented to obtain the desired results [9]. The Artificial Neural Network translates American Sign Language into alphabets. A sensory glove called a smart hand glove with a motion detection mechanism was used for data collection purposes, and as a result, the transmitter-receiver network processed input data to control home appliances and generate recognition results [10]. Hand-body language-based data analysis was performed using a machine-learning approach. A sensor glove embedded with 10 sensors was used to capture 22 different kinds of postures. KNN, SVM, and PNN algorithms were applied to perform sign language posture recognition [11]. The authors in ref. [12] presented a device named the “Electronic Speaking Glove”. This device was developed using a combination of Flex sensors. Flex sensor data were fed into a low-power, high-performance, and fast 8-bit TMEGA32L AVR microcontroller. A reduced instruction set architecture (RISC)-based AVR microcontroller used the “template matching” algorithm for sign language recognition.

Another Sign language recognition-based system was developed by [13]. This virtual image interaction-based sensor system succeeded in recognizing six letters, i.e., “A, B, C, D, F, and K,” and a digit, “8”. So, the prototype was developed using two flex sensors attached to the index and middle finger of the right hand. These sensor data were transmitted towards the Arduino Uno microcontroller. In this experimental setup, MATLAB-based Fuzzy logic was implemented. A sign gesture recognition-based prototype was developed by the authors in ref. [14]. This prototype consisted of a smart glove embedded with five flex sensors. This acquired data were then sent towards the Arduino Nano microcontroller, and a template matching algorithm was used for gesture recognition purposes. This experimental set succeeded in recognizing four gestures made for Sign Language. A Liquid Crystal Display (LCD) and a speaker were used to display and speaking recognized gestures, respectively. The authors in ref. [15] developed an experimental model for Standard American Sign Language (ASL) alphabet recognition. A programmable intelligent computer (PIC) was used to store the predefined alphabet data of ASL alphabets. This experimental setup was also based on template matching phenomena. For data acquisition purposes, a smart prototype based on three flex sensors along with an analogue to digital (ADC) converter, an LCD, and an INA 126 instrumentation speaker were utilized. In this setup, the 16F377A modeled microcontroller was used, which succeeded in recognizing 70% of ASL alphabet gestures.

A more advanced, intelligent, and smart system was implemented by the authors in ref. [16]. Their experimental setup included eight contact sensors and nine flex sensors. These sensors were placed inside and outside of fingers. The outer five sensors were deployed to detect bending changes, and the inner four sensors were attached to measure hand orientation. This system was also based on a template matching algorithm where a unique 36 gesture-based standard ASL dataset was matched with input data. The ATmega 328P microcontroller was used for matching purposes, which succeeded in producing 83.1% and 94.5% accuracy for alphabet and digits, respectively.

In ref. [17], the authors made a Standard Sign language recognition prototype. This prototype consisted of five flex sensors embedded with an ATmega 328 based microcontroller. Senor-based acquired data were compared with the already stored ASL dataset. This experimental set succeeded in producing 80% overall accuracy. To facilitate the deaf-mute community, another important contribution was presented by ref. [18]. The authors succeeded in developing a prototype which translates sign language into its perfectly matched alphabet or digit. Eleven resistive sensors were used to measure the bending of each finger. Two separate sensors were utilized in this scenario to detect wrist bending. The developed smart device worked perfectly on static gestures and produced good results in alphabet recognition with an overall accuracy of 90%. A Vietnamese Sign language recognition system was developed using six accelerometer sensors [19]. This prototype was designed for 23 local Vietnamese gestures, including two extra postures of “space” and “punctuation”. Gesture classification was performed using Fuzzy logic-based algorithms. This device, named “AcceleGlove”, succeeded in producing 92% overall accuracy in Vietnamese Sign language recognition. A posture recognition system was developed in ref. [20]. This innovative glove-based system was assembled using a flex sensor, force sensor, and a MPU6050 Accelerometer sensor. Five flex and five force sensors were attached to each finger, and an accelerometer was attached to to the wrist. The experimental setup comprised data from flex sensors, force sensors, gyro sensors, accelerometer sensors, and IMU sensor data. All these sensors related to Arduino Mega for data acquisition. Based on data classification, the output was displayed on LCD. This system achieved 96% accuracy on average. A real-time sign-to-speech translator was developed to convert static signs into speech by using “Sign Language trainer & Voice converter” software [21]. Data were acquired using five flex sensors and a 3-axis accelerometer sensor connected with an Arduino-based microcontroller.

A handmade sign recognition system was developed with the help of LabVIEW software using the Arduino board. The user interacted with the environment using the LabVIEW provided Graphical User Interface (GUI), and recognition was performed with the help of Arduino [22]. Another smart-sensor-embedded glove was developed by [23]. A good combination of flex sensors, contact sensors, and a 3-axis ADXL335 accelerometer was used for recognition purposes. Flex sensors were attached to each finger of the hand, and contact sensors were placed in between two consecutive fingers. The sign language-based gestures were obtained using a described smart glove. These sign-based analog data were transferred towards the Arduino Mega environment for recognition purposes. Classified sign gestures were displayed with the help of a 16 × 2 Liquid Crystal Display (LCD) and were converted into speech with the help of a speaker. A smart glove based on five flex sensors and an accelerometer was designed for sign language recognition [24]. This data glove transferred analog signal data to the microcontroller for recognition. Lastly, the output was shown with the help of pre-recorded voice matched with a recognized sign. A sign language recognition system based on numeric data was developed in ref. [25]. The authors used a combination of a Hall sensor and a 3-axis accelerometer. The smart data glove was composed of four Hall sensors attached to the fingers only. Hand orientation was measured with the help of an accelerometer, and finger bend was detected by using Hall sensors. These analog sensor data were passed towards MATLAB code to ideally recognize signs made by smart gloves. This experimental setup was only tested on numbers ranging from 0 to 9. The developed system succeeded in producing an accuracy of 96% in digit recognition.

Despite traditional sensor-based bright data gloves, another advanced approach was utilized by [26]. A smart glove for gesture recognition was created by using LTE-4602 modeled light emitting diodes (LEDs), photodiodes, and polymeric fibers. This combination was used only to detect finger bending. Hand motion was also captured using a 3-axis Accelerometer and gyroscope. This portable smart glove succeeded in hand gesture recognition made for sign language translation. The authors also made regional sign language systems from different origins. An Urdu Sign Language-based system was developed in ref. [27]. The smart data glove was composed of five flex sensors attached to each finger, and a 3-axis accelerometer was placed at the palm. To display the output, a liquid crystal display (LCD) of 16 × 2 dimensions was used. The authors succeeded in creating a dataset of 300 × 8 dimensions, and the Principal Component Analysis (PCA) technique was utilized using the MATLAB software to detect static sign gestures. Using PCA, the authors succeeded in achieving 90% accuracy. Another regional sign language recognition system was presented in ref. [28]. The authors made a prototype to convert Malaysian Sign Language. A smart data glove made of Tilt sensors and a 3-axis accelerometer was developed for recognition purposes. Microcontroller and Bluetooth modes were also involved in this prototype to classify detected signs and transmit them to a smartphone. The microcontroller operated on template-matching phenomena and succeeded in recognizing a few Malaysian Sign Language gestures. Overall system accuracy was from 78.33% to 95%. Flex sensor and accelerometer-based smart gloves can perform alphanumeric data classification. Using this prototype, 26 alphabets and ten digits can be recognized using a template-matching algorithm [29]. Five flex sensors attached to each finger produced an analog signal of a performed gesture which was transferred towards an Arduino Uno microcontroller. Including an accelerometer for hand motion detection, the authors obtained eight valued data for a sign gesture. In ref. [30], the authors developed two gloves-based models. These models contained ten flex sensors attached to each finger of both hands and a 9 degree of freedom (DoF) accelerometer for motion detection. Two glove-based systems were tested on phonetic letters, including a, b, c, ch, and zh. With the help of a matching algorithm, the authors performed static sign recognition with approximately 88% accuracy.

American Sign Language classification and recognition-system-based probabilistic segmentation were presented in ref. [31]. This system was divided into two main modules. The first module performed segmentation based on the Bayesian Network (BN). Data obtained during this session were used for training purposes. The second module was based on classification using a combination of Support Vector Machine (SVM) classifier with multilayer Conditional Random Field (CRF). This system succeeded in producing 89% accuracy on average. The authors in ref. [32] brought some innovation in existing sign language recognition systems by combining data obtained from sensor gloves and the data obtained using hand-tracking systems. A very well-known methodology known as Dumpster-Shafer theory was implemented on the obtained and fused data for evidence assembling. This fused system achieved 96.2% recognition accuracy on 100 two-handed ArSL. Hand motion and tilt sensor-based sign data were collected using Cyber Glove [33]. The classification of 27 hand-shapes based on Signing Exact English (SEE) was performed using Fisher’s linear discriminant embedded with a linear decision tree. Vector Quantization Principal Component Analysis (VQPCA) was used as a classification tool for sign language recognition. This system was successful in obtaining 96.1% overall accuracy.

An Arabic Sign Language recognition-based deep learning framework focusing on the singer independent isolated model was discussed in ref. [34]. The main focus of this research was on the regional sign gestures. In a vast variety of regional domains, these authors focused on only Arabic sign gestures and implemented deep learning-based approaches to achieve the desired results. Implementation of hand gestures recognition for posture classification was implemented in ref. [35]. The prototype was purely based on real-time hand gesture recognition. For implementation, an IMU-based data glove embedded with different sensors was used to achieve the desired results. Another advancement in the field of sensor-based gestures recognition was implemented by ref. [36]. A dual leap motion controller (LMC)-based prototype was designed to capture and identify data. Gaussian Mixture Model and Linear Discriminant based approaches were implemented to achieve results. A case study-related implementation based on regional data was implemented in ref. [37]. The authors focused on Pakistani Sign Language models to work on Multiple Kernel Learning-based approaches. Working with signal-based sensor values for classification of real-time gestures was implemented in ref. [38]. The authors worked on wrist-worm-based real-time hand and surface postures. EMGs and IMU-based sensors were embedded to achieve the desired values of sign postures. An armband EMG sensor-based approach was implemented by the authors in ref. [39]. The main focus was to classify finger language by utilizing ensemble-based artificial neural network learning. The sensor values helped ANN to classify gestures accurately. A sign language interpretation-based smart glove was designed by the authors in ref. [40]. A sensor-fused data glove was used to recognize and classify SL postures. Another novel approach to capture sign gestures was discussed in ref. [41]. The developers of the smart data glove named it SkinGest as it completely grips skin with no detachments. For capturing gestures and postures data, filmy stretchable strain sensors were used. Leap motion-based identification of sign gestures was implemented with the help of a modified LSTM model in ref. [42]. Continuous sign gestures was perfectly classified using an LSTM model to get the desired results. Another novel approach of working with key frame sampling was implemented in ref. [43]. The authors also focused on skeletal features to utilize an attention-based sign language recognition network. A Turkish sign language dataset was processed using based line methods in ref. [44]. Large scale multimodal data were classified based on regional postures to achieve good recognition results. Similarly, the authors used Multimodal Spatiotemporal Networks to classify sign language postures in ref. [45]. Development of a low cost model for translating sign gestures was targeted in ref. [46]. The main focus was the development of a smart wearable device with a very reasonable price.

### 2.2. Vision Based Models

The authors in ref. [47] developed a model using vision-sensor-based techniques to extract temporal and spatial features from video sequences. The CNN algorithm was applied on removed lines to identify the recognized activity. An American Sign Language dataset was used for feature extraction and activity recognition. An Intel Real sense camera was used to translate American Sign Language (ASL) gestures into text. The proposed system included an Intel Real camera-based setup and applied SVM and Neural Network (NN) algorithms to recognize sign language [48]. Due to the large set of classes, inter-class complexity was increased to a large extent. This issue was resolved using the Convolutional Neural Network CNN-based approach. Depth images were captured using a high-definition Kinect Camera. Obtained images were processed using CNN to obtain alphabets [49]. Real-time sign language was interpreted using CNN to perform real-time sign detection. This approach does not include outdated datasets or predefined image datasets. The authors manually implemented a real-time data analysis mechanism rather than the traditional approach of using predefined datasets in ref. [50]. In vision-sensor-based recognition, 20 alphabets with numbers were recognized using Neural Network-based Hough Transform [51]. Due to the image’s dataset, a specific threshold value of 0.25 was used for efficiency achievement in the Canny edge detector. This system succeeded in achieving 92.3% accuracy. Fifty samples of alphabets and numbers were recognized by the Indian sign language system using a vision-sensor-based technique [52]. A support vector machine (SVM)-based classifier with B-spline approximation was used, which achieved 91% accuracy on average. A hybrid pulse-coupled neural network (PCNN) embedded with a nondeterministic finite automaton (NFA) algorithm was used collectively to identify image-based gesture data [53]. This prototype achieved 96% accuracy based on the best match phenomena.

Principal component analysis (PCA) along with local binary patterns (LBP) extracted Hidden Markov Model (HMM) features with 99.97% accuracy in ref. [54]. In ref. [55], hand segmentation based on skin color detection was used. For hands identification and tracking, a skin blob tracking system was used. This system achieved 97% accuracy on 30 recognition words. In ref. [56], Arabic Sign language recognition was performed using various transformation techniques like Log-Gabor, Fourier, and Hartley transform. Hartley transform and Support Vector Machine (SVM) and K-Nearest Neighbor (KNN) classifiers helped produce 98% accuracy. Combined orientation histogram and statistical (COHST) features along with wavelet feature techniques were used in ref. [57]. These techniques succeeded in recognizing static signs made for numbers from zero to nine in ASL. The neural network produced efficient results based on the feature values of COHST, wavelet, and histogram with 98.17% accuracy. Static gesture recognition based on alphabets was performed using neural network-based wavelet transform. This system achieved 94.06% accuracy in recognizing Persian sign language [58]. Manual signs were detected using the finger, palm, and place of articulation. Equipment arranged for manual sign extracted data from a video sequence and matched it with a 2D image of standard American Sign Language alphabets. The proposed setup resulted in accurate sign detection of alphabets [59].

Deep learning-based SLR models are also focused on vision-based approaches. The authors in ref. [60] focused on current deep learning-based techniques, trends, and issues in deep models for SL generation. Keeping in mind standard American Sign Language models, the authors in ref. [61] focused on the development of a deep image-based user independent approach. Their main work was based on PCANet features based on depth analysis. Another edge computing-based thermal image detection system was presented by the authors in ref. [62]. They worked on digit-based sign recognition model using deep learning approaches. Different computer vision-based techniques were applied for SLR tasks. A camera sensor-based prototype was used by the authors in ref. [63] to correctly identify sign postures. A convolutional neural network-based approach was implemented by using video sequences in ref. [64]. A three-dimensional attention-based model was designed for a very large vocabulary to acquire data from video sequences and classify them using a 3D-CNN model. Similarly, the same authors implemented a boundary adaptive encoder using an attention-based method on a regional Chinese language dataset in ref. [65]. A novel key-frame-centered clip-based approach was implemented on the same Chinese Sign Language-based dataset in ref. [66]. The regional Chinese sign dataset was classified using video sequences in the form of images. This vision-based novel approach produced challenging results in CSL. Another fingerspell-based smart model was developed by the authors in ref. [67]. They focused on the development of an Indian quiz portfolio that was based on camera-oriented posture classification. The main point of identification was based on ASLR models using a vision-based approach. A vision sensor-based three-dimensional approach was implemented by the authors in ref. [68]. Three-dimensional sign language representation was classified with the help of spatial three-dimensional relational geometric features. These 3-D data were classified and recognized with the help of a S3DRGF-based technique quite efficiently. Another vision-based technique focusing on color mapping-based classification and recognition was developed by the authors in ref. [69]. A CNN-based deep learning model was trained on the three-dimensional data of signs. Color texture coded-based joint angular displacement maps were classified efficiently with the help of a 3-D deep CNN model. Another advanced approach based on three-dimensional data manipulation for sign gestures was implemented in ref. [70]. The authors focused on classification and recognition of angular velocity maps with the help of the deep ResNet model. Connived Feature ResNet was deployed specifically to classify and recognize 3-D sign data. Another video sequences-based novel approach to classify sign gestures was implemented in ref. [71]. A BiLSTM-based three-dimensional residual neural network was used to capture video sequences and identify the posture data. A novel deep learning-based hand gesture recognition approach was implemented by the authors in ref. [72]. Image-based fine postures were captured and perfectly recognized using deep learning-based architecture. A virtual sign channel for visual communication was developed in ref. [73]. The authors’ main focus was to create a virtual communication channel for deaf-mute and hearing individuals. Another three-dimensional data representation for Indian sign language was developed in ref. [74]. The authors used an adoptive kernel-based motionlets-matching technique to classify gesture data. A video sequence and text embedding-based continuous sign language model was implemented in ref. [75]. Joint latent spaces-based data were processed using cross model alignment of a continuous sign language recognition model.

### 2.3. Non-Commercial Models for Data Glove

In non-commercial systems, most authors work on finger bend detection regarding any sign made. So, a large variety of different solo sensors or a combination of different sensors were used to detect this finger bending. The authors in ref. [76] developed a non-commercial-based prototype for sign language recognition. This system was completely based on the finger bending method. To detect finger bending, ten flex sensors were used. A pair of sensors were attached to two joints of each finger. To deal with analogue flex data, a MPU-506A multiplexer was used. Selected data coming from the multiplexer were sent to the MSP430G2231 microcontroller. A Bluetooth module was used to transmit data towards a smart cell phone. This captured data were then compared with the sign language database and the sorted result was converted into speech using a text-to-speech converter. The authors in ref. [77] also succeeded in developing a non-commercial sign language recognition-based prototype. This prototype included five ADXL 335 accelerometer sensors connected with an ATmega 2560 microcontroller system. Based on axis orientation, sign language was identified and transmitted via a Bluetooth module towards mobile application for text-to-speech conversion. In ref. [78], a prototype was developed to help handicapped people. This prototype converted finger orientation into some actions. For this purpose, five optical fibers sensors were used to collect finger bending data. These 8-bit analog data were used to train multilayered neural networks (NN) using MATLAB. So, six hand gesture-based operations were performed using the Backpropagation training algorithm. For data validation, a tenfold validation method was implemented on 800 sample records. Similarly, for Sign Language Recognition, the authors made a non-commercial prototype based on five flex sensors [79]. The MSP430F149 microcontroller was used to classify incoming analog data. These data were compared with standard American Sign Language (ASL) data, and the output was displayed on Liquid Crystal Display (LCD). Using text-to-speech methodology, the recognized letter was converted into speech using a good quality speaker. The authors in ref. [80] developed the Sign-to-Letter (S2L) system. This system contained six flex sensors and a combination of discrete-valued components and a microcontroller. Five flex sensors were attached to five fingers of the hand, and one sensor was attached to the wrist of the same hand. This combination of two different bending-based sensors succeeded in converting signs into the letter. The output of this system was displayed via the programming “IF-ELSE” condition. A combination of Light Emitting Diode- Laser Dependent Resistor (LED-LDR) sensors was used by [81]. MSP430G2553 microcontroller was used to detect signs made by finger bending. Using mentioned microcontroller, analog data were converted into digital and ASCII codes related to 10 Sign Language Alphabets. Converted data were transmitted using a Bluetooth module named as ZigBee, and recognized ASCII code was displayed on a computer screen. This code was also converted into speech.

Another fingerspell system was developed in ref. [82]. This prototype included four flex sensors and an accelerometer sensor. The main idea in this prototype design was to translate handmade signs into their corresponding American Sign Language (ASL) alphabets. For data acquisition, four deaf-mute individuals were gathered. This system succeeded in understanding 21 gestures out of 26. A hand gesture recognition system was developed by measuring inertial measurements along with altitude values [83]. For data acquisition, six Inertial Measurement Units (IMUs) were used in this prototype. Each IMU was attached to each finger, and one IMU was attached to the wrist. This experimental setup succeeded in collecting hand gesture data by an accelerometer and a gyroscope, and a magnetometer sensor provided values. These values were refined using Kalman Filter and processed through the Linear Discriminant Analysis (LDA) algorithm. Overall, 85% accuracy was achieved by using this prototype in hand gesture recognition.

### 2.4. Commercial Data Glove Based Models

Besides following the traditional way of making cheap data gloves, some of the authors used a commercial data glove named “CyberGlove”. This commercial glove was specifically designed for deaf-mute people. A lot of affected communities and research centers used this glove for communication and research purposes. CyberGlove was manufactured precisely with the combination of 22 sensors embedded on the glove. The basic structure of the glove contained four sensors attached in between fingers and three sensors attached on each finger. Palm sensors and wrist bending measurement sensors were also included in this commercial prototype. This smart, thin layer, elastic fiber-based sensor glove had an approximate cost of $40,000 for each pair. Using this CyberGlove, authors in ref. [84] applied a combination of neural network-based algorithms to measure the accuracy and efficiency of the system. Finger orientation and hand motion projection were captured with a smart CyberGlove embedded with a 3D motion-tracker sensor. This analog signal data were transferred towards a pair of word recognition network and velocity network algorithms. These algorithms worked on 60 American Sign Language (ASL) combinations and obtained an accuracy of 92% and 95%, respectively. A posture recognition system based on a 3D hand posture model was developed in ref. [85]. A Java 3D-based model helped in classification and segmentation of real-time input posture data. These data were compared with pre-recorded CyberGlove-based data with the help of an index tree algorithm. Another CyberGlove embedded with a 3D motion tracker named as Folk of Birds was used for sign language recognition. CyberGlove-based data containing bend, axis, motion, and hand orientation were fed into the multilayered neural network. The Levenberg-Marquardt backpropagation algorithm was used for segmentation and sign classification. This prototype succeeded in producing 90% accuracy in American Sign Language (ASL) recognition [86].

In the sensor-based sign language recognition domain, another advancement was made by introducing a new five-dimensional technology commercial data glove commonly known as the 5DT data glove. This 5DT commercial glove was made in two variants, one with five fiber optic sensors and the other with fourteen optic sensors. 5DT manufacturers named this fiber optic smart data device ultra-motion. Internationally this data glove’s cost was approximately $995. In five sensor-based data gloves, each optical sensor is attached to each finger, and one sensor is attached for hand orientation detection. In 14 optic fiber sensors, two sensors are kept in contact with one finger, and a sensor is also attached in between fingers to check finger abduction. Two-axis measurement-based sensors are also attached in that glove to determine axis and orientation, including pitch and roll of the hand. So, these 5DT-based bright data gloves were used by authors for Japanese Sign language recognition [87]. The main idea of developing this system was to automate the learning system. A 3D model based on the 5DT 14 sensor-based smart data glove for simulating signs was made. This system highlights motion errors for beginners and helps understand hand motion completely via a 3D model. To facilitate communication for deaf and mute people, another advancement was applied using a combination of 5DT data gloves with five embedded sensors. Data obtained by using ultra motion glove were trained using the MATLAB simulator. A multilayered neural network with five inputs and 26 outputs was utilized for the training model for sign language recognition. A series of NN-based algorithms like resilient, back, quick, and Manhattan propagation, including scaled conjugated gradient, was used for the training model [88].

Another advancement in sign language recognition was seen in ref. [89]. The authors used a DG5 V hand data glove for data acquisition. The internal structure of the DG5 V hand data glove contained five flex or bending sensors with one three axis accelerometer and three contact sensors. This data glove was capable of transmitting acquired data wirelessly. The overall system was made remotely functional by using a battery. The DG 5 V commercial data glove was used for American and Arabic Sign language recognition systems. The authors focused on Arabic Sign Language, whereas this data glove had already been used previously for American Sign Language. The only left-hand glove cost $750. A pair of DG5 V data gloves were used in Arabic Sign language recognition. Two glove-based models succeeded in acquiring data for 40 sentences. This dataset was classified using a modified K-Nearest Neighbor (MKNN) algorithm. The overall system succeeded in producing 98.9% accuracy. The hand gesture cannot be fully recognized without knowing hand orientation and posture. Therefore, an advancement in the traditional system was brought by fusing the concept of Electromyography and inertial sensors within the system [90]. Using a combination of the Accelerometer (ACC) sensor with Electromyography (EMG), the authors achieved multiple degrees of freedom for hand movement. This setup was used for Chinese Sign language recognition. The EMG sensors were attached at five muscle points over the forearm, and the MMA7361 modeled 3-axis accelerometer was attached over the wrist. Multi-layered Hidden Markov Model and decision tree algorithms were used for recognition purposes, which succeeded in producing 72.5% accuracy.

The same setup of Accelerometer and Electromyography was used for German Sign Language. The authors used a single EMG with a single ACC sensor to recognize a small database of German vocabulary. The training was performed on seven words with seventy samples for each word. K-Nearest Neighbor (KNN) and Support Vector Machine (SVM) classifiers were used. The system succeeded in achieving an average accuracy of 88.75% and 99.82% in the case of subject dependency [91]. A similar hybrid approach of Accelerometer and Electromyography was used for the Greek Sign language recognition system. The experimental setup consisted of five-channel Electromyography and an accelerometer sensor. The experiment was conducted on the singer with the intrinsic entropy mode. Experiments repeated ten times on three native singers produced training data. So, the system was trained using the intrinsic entropy mode on MATLAB. The system’s overall accuracy was 93% collectively (without the personal effect of native singers involved for data collection purposes) [92].

### 2.5. Hybrid Recognition Models

A vision-sensor-based approach was also adopted in sign language recognition. The previously used combination of electromyography with an accelerometer was replaced with a vision-sensor-based hybrid approach. In the hybrid approach, the authors used a variety of accelerometers with vision-sensor cameras. The purpose of a hybrid system was to enhance data acquisition and accuracy. The vision-sensor-based hybrid prototype contained red, green, and blue (RGB) color model cameras, depth sensors, and accelerometer-based axis and orientation sensors. This combination of the smart hybrid approach was used for gesture identification purposes. The experimental setup included seven IMU accelerometer sensors attached to the arm, wrist, and fingers. For data acquisition, five different age group sign language speakers performed ten times repeated forty gestures. Parallel Hidden Markov Model (PaHMM) succeeded in producing 99.75% accuracy [93]. Another combination of an accelerometer-based glove and camera sensor was used for American Sign Language recognition. The experimental setup contained a camera attached to a hat for detecting correctly made signs. Nine accelerometer sensors were used for gesture formation: five attached on each finger and two on the shoulder and arm to detect arm and shoulder movement. Two sensors were attached to the back of the palm for hand orientation measurement. This setup was tested on 665 gestures using the Hidden Markov Model (HMM) and produced a per sign accuracy of 94% [94].

### 2.6. Framework-Based Recognition Models

Most of the articles [95,96,97,98] followed a predefined framework for sign language recognition. The main objective of using the same framework was to enhance data accuracy and dataset efficiency. The authors in ref. [99] correctly developed a sign language system and implemented that system using different classification and recognition algorithms. The authors in ref. [100] succeeded in creating a Vietnamese Sign Language framework that worked wirelessly. A two-handed wireless smart data glove was designed and developed using bend and orientation measurement. The experimental setup included MEMS accelerometer sensors attached just like the Accele Glove and as an addition one more sensor was attached to the palm of hands for orientation measurement. Wireless communication was made feasible by using a Bluetooth module attached to a cellphone. The user-generated sign was compared with the standard sign language database, and the correctly found result was displayed on a cellphone screen. Finally, a text-to-speech Google translator was utilized to convert the recognized sign alphabet into speech. This sign language framework succeeded in producing a reasonable accuracy. Similarly, the authors in ref. [101] developed an Arabic Sign Language recognition system. The main purpose of developing another framework for static sign analysis was to minimize the number of sensors on data gloves. This experiment was simulated on the Proteus software. The two-handed glove system contained six flex sensors, four contact sensors, one gyroscope, and one accelerometer sensor on each hand.

Another algorithmic-based sign language recognition framework was designed in ref. [102]. Stream segmentation-based sign descriptors and text auto-correction-based algorithm were utilized. The system also provided software architecture of descriptors for hand gesture recognition. The Sign Language-based Interpolator, which converted text into speech, was also designed in ref. [103]. The overall system framework contained four basic modules that included the smart data glove, training algorithms for the input sign dataset, wirelessly visible sign application, and sign language database for matching the input sign with the standard repository. A very simple resistor-based framework was developed and implemented by ref. [104]. The authors used ten resistors and detected finger movement only. This was a medical application used only for finger flexion and extension. This was a very simple, low-cost, efficient, reliable, and low-power trigger. A data glove containing resistor-based framework was directly connected with a microcontroller which further transmits captured data towards a computer for finger movement analysis. Another simple gesture recognition-based framework was developed by ref. [105]. The smart spelling data glove consisted of three bending sensors attached on three fingers. The authors worked only on five gestures, including thumbs-up and rest. Input gesture data were fed into the microcontroller for recognition purposes, and analyzed gestures were combined in a row to form meaningful data before transmitting them to the receiver. A detailed review on all the frameworks based on Chinese Sign Language was discussed in ref. [106]. All the technical approaches that are only related to the regional Chinese Sign Language recognition and classification mechanisms were discussed in detail. Another detailed review on all the wearable frameworks and prototypes related to sign gesture classification was discussed in ref. [107]. The authors focused on maximum frameworks that are related to and had been previously used by authors in the same field. This is also a review article with good depth of technologies and frameworks in SLR.

## 3. Components and Methods

**Figure 4:**
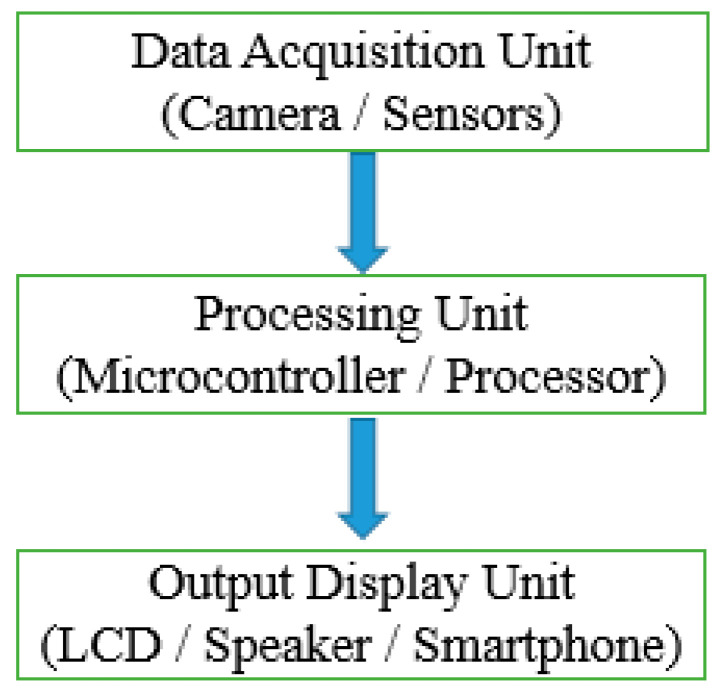
Sign recognition components.

This section emphasizes different methods of prototype formation and techniques used to perform sign recognition. In prototype formation, the developer’s main focus is to design such a system that effectively finds a solution for the current problem. So, there are two main classes for prototype formation. One is different sensor-based, and the other is vision-sensor-based. The hybrid approach is also the combination of these two main approaches. Considering the vision-sensor-based approach, here, the prototype contains a camera as sensor for gesture detection and a CPU for internal algorithm processing. For different sensor-based systems, the input is captured with a sensor data glove for sign detection and a CPU with some machine learning-based algorithms. The output in both cases is the same, which can be any monitor, liquid crystal display (LCD), or any analysis window for operational observations. Mainly, sign language recognition systems can be divided into three main categories. One is the data acquisition process where sign data in any format, either by camera or by combination of sensors, are obtained. Secondly, the processing of acquired data. This step includes a microcontroller board or processor for data processing purposes. Thirdly, the display of results obtained after data processing. This display can be any monitor, speaker, smartphone, or LCD with sign language translated into some meaningful information. The whole system is represented in Figure 4 given below.

### 3.1. Data Acquisition Unit

The data acquisition unit is considered the most important unit of the sign language recognition system. Sensor-based or vision-sensor-based approaches use cameras and combinations of sensors for data acquisition purposes, respectively. In comparison, the hybrid approach uses both sensor and camera for acquisition purposes. For the vision-sensor-based approach, high-definition Kinect cameras are mostly used, and for the sensor-based approach, different sensors like flex, gyro, or tactile are used.

**Figure 5:**
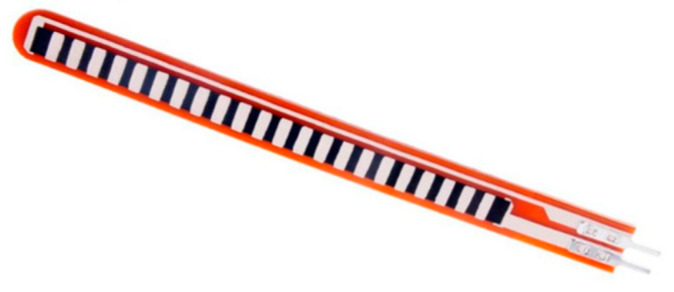
Flex sensor or bend sensor [12,14,26].

Bending Sensor: Considering sign language systems, it is clearly observed that finger bending plays a vital role in gesture formation. This is the reason that such sensors are mostly used, which provide efficient results related to finger bending. The flex sensor shown in Figure 5 is the most helpful sensor in sign recognition as it provides a good effect of figure bending [12,14,26].

Considering the internal structure and working of the flex sensor, it is made of resistive material like carbon. They are very flexible, lightweight, and easily attachable. Internationally, the flex sensor is made in two versions: one with 2.2-inch length and the other with 4.5-inch length, with the market price ranging from $15 to $20 approximately. Both sensor versions are application-dependent and are used accordingly. Initially, when no bending is performed, it produces an average resistance value depending upon the material. The value of resistance increases as more bending is performed. Thus, the degree of bend and resistance value is directly proportional to each other. Typically the resistance of the flex sensor lies in the range of 30 k to 40 k. This sensor works on the voltage division rule shown in Figure 5. Flex sensor resistance is divided by the sum of flex sensor resistance and normal resistance embedded in the circuit and multiplied by voltages in the circuit.

Another important sensor in gesture recognition is the optical sensor. This is a sensor that converts incident light rays into electrical pulses or signals. The purpose of using this type of sensor is to detect finger bending angles. This sensor is mainly used in an environment where an indicated person is unable to move their fingers. Thus, the generated electrical signal helps in completing some specified tasks. Incident light produces different electrical signals for different bending orientations of the fingers. Like a flex sensor, this is also a material-dependent sensor. The technology used in sensor formation depicts the amount of incident light [87]. An advanced approach for bend detection is obtained by using a light-emitting diode (LED) and a light-dependent resistor (LDR) together. A light-dependent resistor produces variable resistance whenever an intensity light falls on it [81]. So, in case of no bending, a very low value is received, and when a finger is bent with some angle, it is detected using this sensor combination.

**Figure 6:**
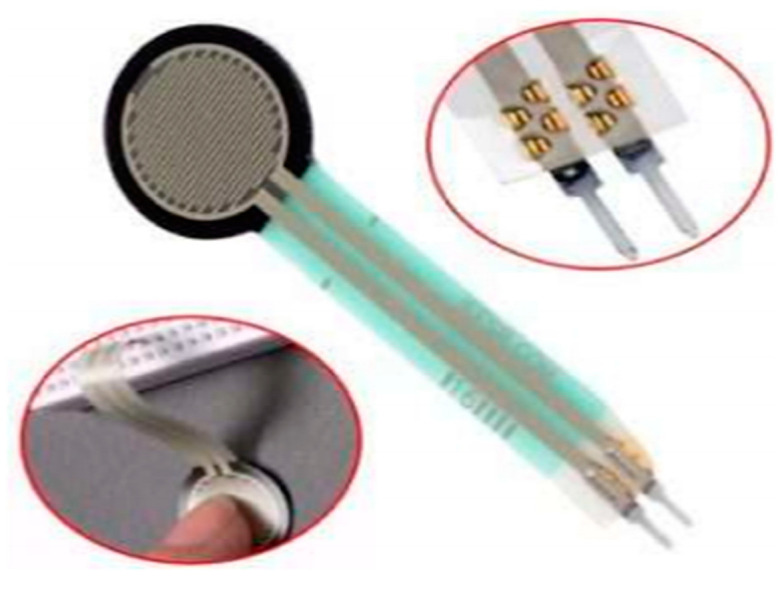
Tactile sensor or force resistive sensor [101].

Another resistive force sensor, known as the tactile sensor, shown in Figure 6, is used normally for sign recognition. This sensor is made of thick polymer material whose resistance can vary. So, whenever a force with some value is applied to the surface of the tactile sensor, its resistivity changes due to its structural material. Similarly, whenever pressure is applied on the material surface, the force applied can also be measured ranging from 1000 N to 100,000 N. As sign language is a combination of different hand and finger orientations, pressure and force values can vary accordingly. There are some orientations when no force is applied to the tactile sensor. In that case, the circuit acts as an open circuit. So, when pressure on the tactile surface is increased, its resistance decreases due to the materialistic property of the sensor [16]. Considering the working mechanism of the sensor, one can easily detect finger bending due to pressure applied on the sensor and can also measure finger orientation and angle position [23,87]. Considering the cost and size of the sensor, it is easily available globally with varying costs from $6 to $25. Size varies according to the price and experimental usage of the sensor. There are different versions of tactile sensor sizes in which 1, 0.5, and 0.16 inches of diameter are normally available.

**Figure 7:**
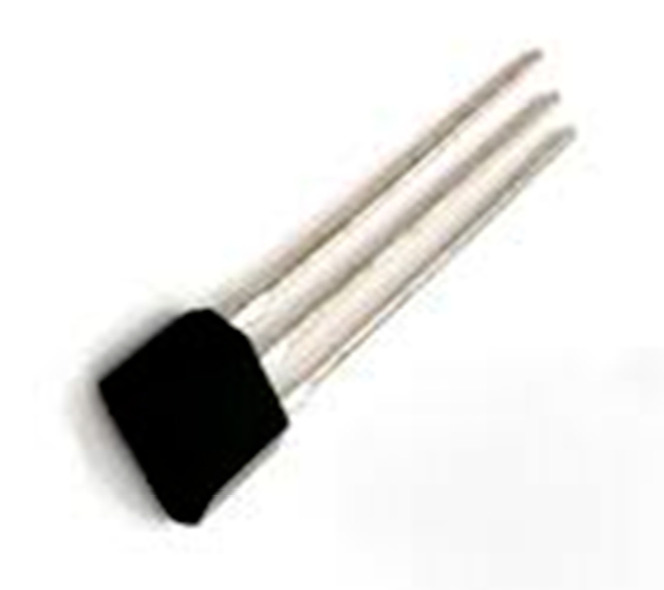
Smart glove with Hall sensors connected to the fingertip [25].

Figure shape and orientation can also be detected using an applied magnetic field. This principle uses voltage variation values obtained from electrical conductor material. This prototype is known as the Hall Sensor. So, as the name suggests, the Hall Effect Magnetic Sensor (HEMS) given below in Figure 7 uses an applied magnetic field to measure voltage variations across the conductor. A lot of versions of the Hall sensor are used for effect measuring purposes, but mostly the unipolar MH183 is normally used. This unipolar magnetic sensor finds the relevant South Pole to perform voltage variation tasks. This lightweight sensor is placed on the tip of each finger. To measure variations, a good capacity magnet is placed onto the palm with the South Pole faced upward. When the magnet sensor is brought in front of the palm magnet, voltage variation ranging from 100 to 400 mV is generated. This prototype is known due to its high level of accuracy.

**Figure 8:**
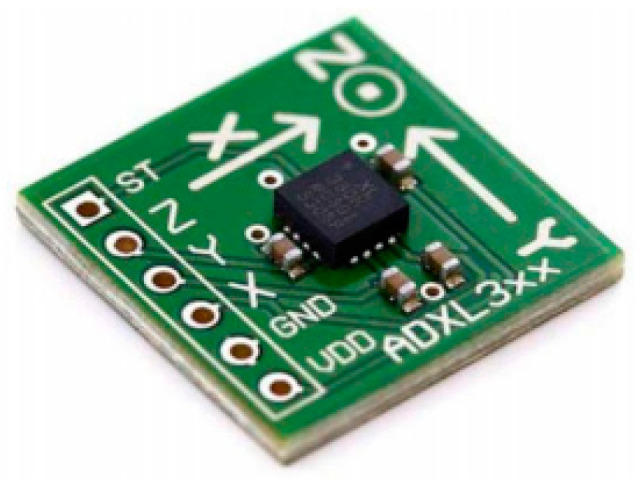
The ADXL335 3-axis ACC with a three-output analog pin x, y, and z [19,20,21].

Some of the articles used the accelerometer ACC sensor to determine the shape and orientation of fingers [19,20,21,77,82,91]. If bending is the only target to be achieved, then only the flex sensor can deal with the problem. However, in sign language, hand movement and orientation are also considered to understand any sign perfectly. In the above-discussed sensors, orientation information cannot be captured. Therefore, an accelerometer sensor is used to determine the hand orientation for sign language recognition. The accelerometer works on Degree of Freedom (DoF) principles. This sensor has axis information along with different acceleration values provided by the sensor. This sensor is also embedded in a smart glove to perfectly distinguish signs made by the user. There are a lot of versions of accelerometer sensors that are used accordingly depending upon the situation to be faced. Normally for axis and orientation purposes, the ADXL335 version given below in Figure 8, made by Adafruit Industries in New York, is used. ADXL335 produces three-axis acceleration and three angles orientation. Physically, the device is very lightweight, small, thin in shape, and low power consuming. Depending upon the functionality and size of the sensor, its price ranges from $10 to $30. ADXL335 uses applications based on tilt-sensing to measure gravity acceleration statically. Dynamically, acceleration is measured from such devices that have vibrating, shocking, and motion effects. Talking about the structure of ADXL335, it is a silicon-based microstructured component. It is made of a silicon ship where a silicon wafer provides resistance values regarding applied forces of acceleration. An accelerometer also provides deflection values. So, deflection is measured using a capacitor that is attached to a moving object. Deflection variation results in changed capacitor values. These changes values produce output sensor values named acceleration.

**Figure 9:**
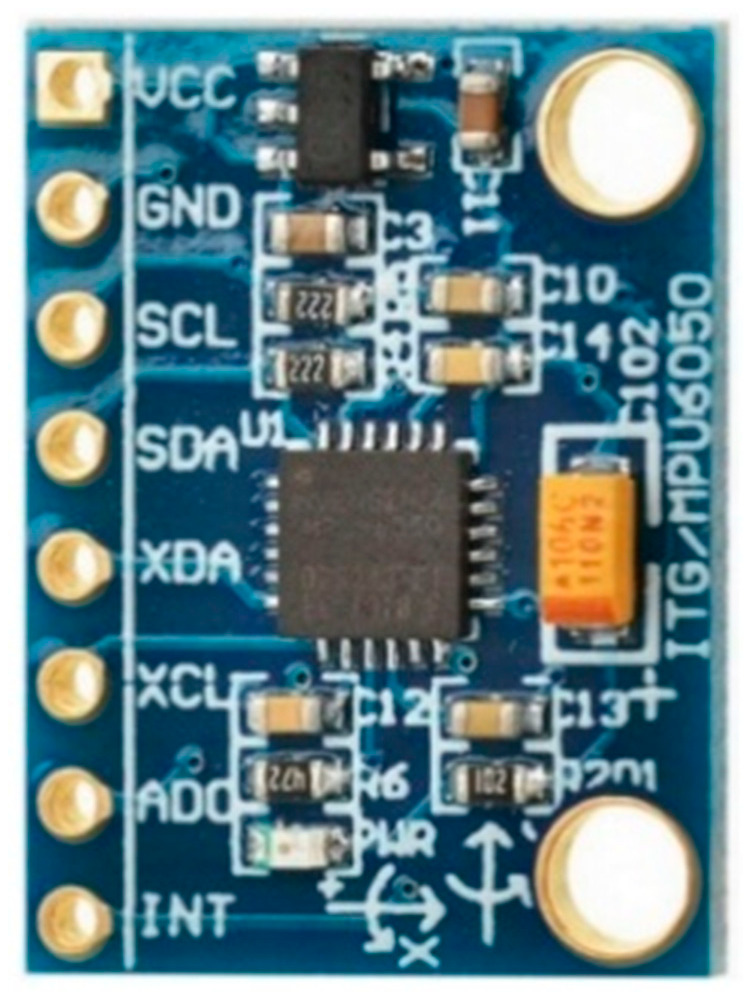
The six DoF IMU MPU6050 chip consists of a 3-axis ACC and 3-axis gyroscope [20,35,38,83].

As discussed above, the inertial measurement unit (IMU) works on six degrees of freedom (DoF). This sensor provides three directional acceleration and three directional orientation. In some applications, only the accelerometer is not enough to cope with motion-related tasks. Rather, the unaffected gravity gyroscope provides help to acquire the best motion-related results [20,35,38,83]. Therefore, the gyroscope and accelerometer are embedded into a single chip for the best and most efficient results, as shown in Figure 9 below. For the processing of tasks, a micro processing unit (MPU) is used. Among a variety of MPUs, the most famous is MPU 6050, which is available internationally at prices ranging from $25 to $30. The MPU 6050 is a six-DoF IMU device that operates on 3 V to 5 V with 400,000 Hz frequency using I2C communication. These data communication values are received with the help of 16 bit analogue-to-digital converter embedded into a micro controller unit (MCU).

**Figure 10:**
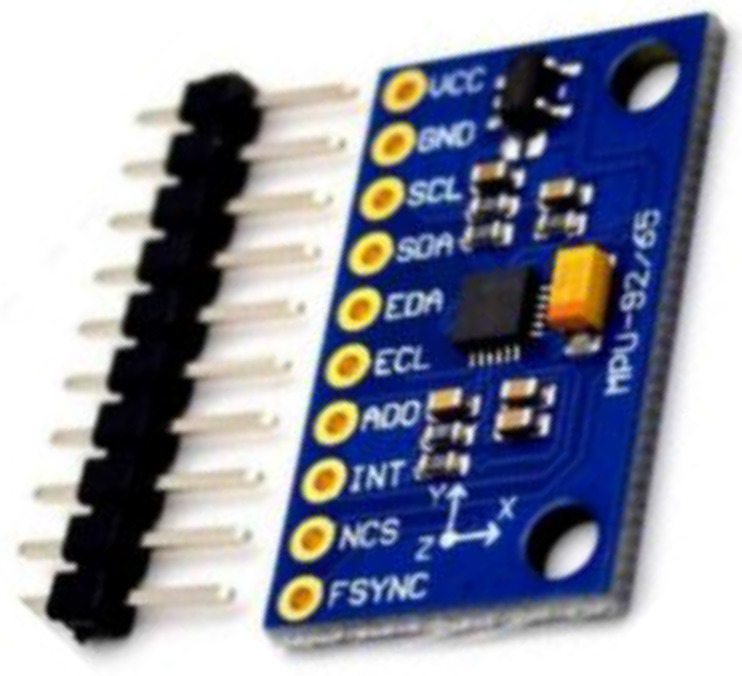
The 9 DoF IMU, MPU-9250 breakouts [30].

Another updated IMU 6050 was obtained by adding the three-axis magnetometer effect on the existing six DoF IMU. This new version is known as the MPU 9250, with prices ranging from $15 to $20. This new device is now based on a microelectromechanical system having the ability to detect and operate on magnetic fields. MPU 9250, given below in Figure 10, provides a three-axis gyroscope, three-axis accelerometer, and three-axis magnetometer. Using 9 DoF IMU, hand orientation and movement for sign language gestures can easily be analyzed. MPU 9250 version of IMU proved better than its previous versions, including MPU 9150 in energy and power consumption.

### 3.2. Processing Unit

**Figure 11:**
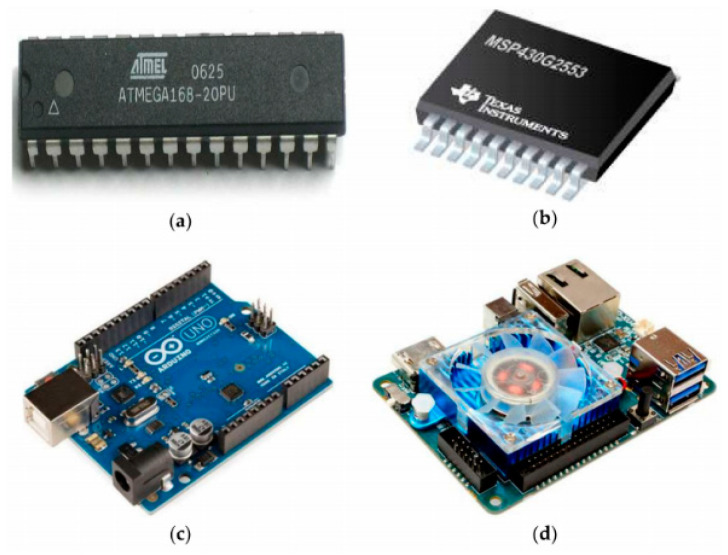
(**a**) ATmega microcontroller, (**b**) MSP430G2553 microcontroller, (**c**) Arduino Uno board, and (**d**) Android XU4 minicomputer [17].

Machines have a central module working as the brain of the system. The processing unit is known as the brain or the system’s mastermind, which performs complete processing tasks from data acquisition to display of results. Acquired data are processed and recognized accordingly by the processing unit. This processed data, which are any sign data, are displayed as outputs using output ports of processing units. Processing units play a vital role in the development of prototypes designed for sign language recognition. A variety of processing units starting from small microcontroller-based chips to complete processing boards has already been used. From the literature review, ref. [12] used an ATmega AVR-based microcontroller chip shown in Figure 11a given below. Atmega is a Reduced Instruction Set Architecture (RISC)-based 8-bit AVR microcontroller. Both memories read and write operations are performed using 32KB flash memory. Similarly, ref. [37] used an 8-channel based 10-bit Analogue to a Digital convertor (ADC), normally named MSP430G2553, as shown in Figure 11b given below. Ref. [12] used ARM7 and ARM9-based microcontrollers for processing purposes. Refs. [13,14,20,21,22,23] used the most common, open-source processing unit known as Arduino-based microcontroller shown in Figure 11c given below. Arduino has a lot of versions in the market. Among which Arduino Nano, Uno, and mega are the most used microprocessors. Based on the literature review analysis, ATmega328P model-based Arduino Uno is commonly used. This Arduino version communicates with the USB port system and operates on 16M Hz quartz crystal frequency. For getting sensor values and displaying output data values, Arduino has six Analogue pins and 14 digital input/output pins. Ref. [17] used a four-CPU core-based Samsung Exynos5 Octa 5410 processor with each core consisting of Cortex A-15. The abovementioned microprocessor is named Android XU4, as shown in Figure 11d given below. This microprocessor is produced by Hardkernel2 and manufactured in South Korea.

### 3.3. Output Unit

The final section in the sign language recognition system is based on the monitoring or output analysis unit. The sign language recognition journey starts from acquiring sign language data, processing the acquired data to extract useful information, and monitoring the desired result using the appropriate output unit. For output analysis purposes, researchers used some sort of display. Researchers including refs. [81,100] used computer or laptop screens for result analysis. Some authors and prototype designers, including refs. [14,15,20,23], used liquid crystal display (LCD) for results analysis. Some authors have used speakers for text-to-speech analysis [93]. Authors using both LCD and speakers are ref. [27]. To send analyzed data in the form of recognized gestures, some authors have used smartphones [28]. They have used data transmission mechanisms from the computer after algorithmic processing and reception of recognized results on smartphone screens. All the above mentioned techniques have been used by authors until now.

### 3.4. Gesture Classification Method

The role of software is very important in any field where data analysis or simulation-based results are needed. The main functionality of the software is to provide a platform where input data can be processed using the best and efficient techniques. These applied techniques will help to produce desired results. So, using good software, methods normally named as algorithms are developed. In the case of sign language recognition (SLR), normally classification, template matching, static posture matching, long term fuzzy logic, and artificial neural network (ANN)-based algorithms have been used. In static posture matching, new incoming data in the form of testing data are matched with pre-defined or trained data using data statistics. Based on classified statistics, data related to the class of best match are assigned accordingly. This is also known as template matching because data used by classification algorithms make a classification template for new incoming testing data [16,25]. The only complication in template matching is the increased number of classes and complex computations.

Introducing the concept of machine learning in the classification process results in a good recognition approach. Among the domain of artificial intelligence, neural networks are used as the best classifiers. Artificial Neural Network (ANN)-based classifiers have been succeeded in both static and dynamic gesture recognition [39]. Data acquisition using data glove and classification obtained using machine learning-based artificial neural networks also produced good results in posture classification [84].

Falling in the domain of ML-based ANN, Discriminant Analysis based on dimensionality reduction proved to produce less complicated and efficient results. Using improved clustering, linear discriminant analysis (LDA) succeeded in producing accurate classification results [41]. Another ML-based classification algorithm includes Hidden Markov Models (HMMs). HMM succeeded in speech recognition, gesture recognition, and posture recognition tasks [54,93,94]. In the sign language recognition domain, K-Nearest Neighbor (KNN) produced good results for American Sign Language (ASL) recognition. Integrating different ML-based algorithms like a combination of HMMs with K-Nearest Neighbor (KNN) produced accurate results of hand gesture classification. Similarly, the combination of KNN with Support Vector Machine (SVM) succeeded in producing better posture classification results [56,89]. Fuzzy logic is normally used in binary classification. However, whenever human interaction is involved in the form of decision making, long-term fuzzy logic is used for wide classification. In Sign language recognition, many classes have to be interacted using long-term fuzzy logic recognition [13,19].

### 3.5. Training Datasets

Classification is the process of a training model to classify testing data. The factors that affect the accuracy of any system include data capacity for training purposes. Many training data are directly proportional to grater classifier accuracy. Choosing a perfect training dataset is also another challenge, which is a subjective matter. A detailed analysis can be written on the types, size, properties, audience and specifications of datasets. In this section, a generalized overview of different datasets is discussed. To obtain the perfect training data, a sensor-based glove is used. This is since sign language is the combination of different fingers orientations. So, any orientation made would produce some sensor values. These sensor values collectively act as training data for classifiers. In a sign language recognition system, training datasets are classified into different domains. Some authors, including refs. [16,23], used a very small number of alphabets of sign language for recognition purposes. Different regions have different types of sign language. So, gesture variation based on region produced different datasets. Region-wise word-based signs are produced by the authors in refs. [19,27,32,76].

Some researchers have extended the work and included standard alphabets and numbers of sign language. Words, numbers, and alphabet-based datasets were used by ref. [37] and succeeded in developing a standard system of sign language. Some authors used already available datasets for sign language and extended work already work by purifying and classifying available sign language datasets [83,87]. Initially, researchers developed a system only to recognize sign language numbers [25,28].

**Figure 12:**
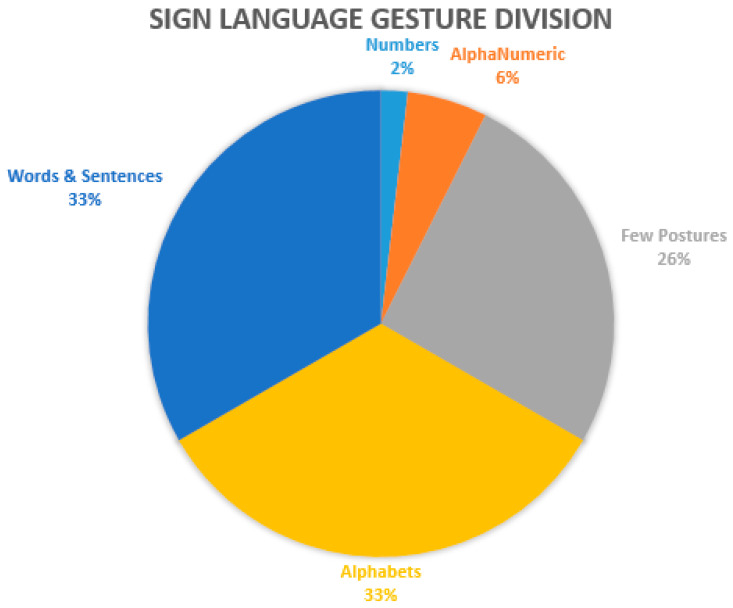
Number of articles on each variety of gestures.

Refs. [77,90,101] worked on numbers and alphabets and extended the training dataset. Mapping a standard sign language system towards daily routine problems was another challenging task. Based on region-wise spoken words, sentences, and phrases, gestures made were converted into meaningful information. These real-time-based sentences covered shopping, greetings, sports, and educational domains. Based on literature review analysis, studied literature is divided into different categories based on experimental domains. Figure 12 given below provides an analysis of the number of articles of prescribed domain like numbers, numbers and alphabets, real-time postures, alphabets and words or phrases with real-life spoken sentences, etc.

Lastly, considering the modern tools and technologies of today’s era, new datasets have been used for the recognition of complex sign postures. Recent studies have clearly mentioned the utilization and importance of these complex datasets for the understanding of complex postures. Despite traditional regional, local, small, and standard ASL datasets, there are some other advanced datasets that have been used by many researchers of the current times. The DEVISIGN-D dataset is one of the mentioned datasets that contains data of 500 daily vocabularies. Data cover eight different signers where vocabularies are recorded twice for four signers, i.e., two male and two female. The total dataset includes 6000 vides [71]. Similarly, the SLR-DATASET contains 25,000 labeled video instances with more than 100 h of data recording time. During dataset collection, 50 different signers were involved in the collection process. Manually, the dataset was testified from Chinese Sign Language professionals [68].

For Chinese Sign Language, two isolated sign language word datasets were used [65]. ID1 is the public large-scale vocabulary Chinese sign language, CSL, a dataset that contains 125k samples consisting of 500 sign words, each of which was recorded five times by 50 signers; the larger scale vocabulary dataset ID2 was collected by the authors using the Microsoft Kinect 2.0 device for CSL. The ID2 dataset is, in its turn, divided into two parts, ID2-spit1 and ID2-spit2 (specifically, ID2-split1 contains 50k samples consisting of 500 sign words, each of which was recorded 10 times by 10 signers; and ID2-split2 contains 20k samples consisting of 2000 sign words, each of which was recorded one time by one signer) and one continuous sign language sentence dataset CD which contains 25k videos consisting of 100 different sign language sentences; each sentence was recorded five times by 50 sign speakers; the length of each video is 4~8 sign words, which is about 15 s.

Finger spelled Indian Sign Language (ISL) signs were captured for training the model used for sign recognition. Capturing was carried out through mobile cameras, laptop cameras, and digital SLR’s. Signs corresponding to 20 finger-spelled alphabets were captured. This was collected with the help of 15 signers: six male and nine female. Close to 1500 images were collected for each sign, making the total number of images collected to about 20 × 1500. Among the captured signs, certain alphabets like “A” and “B” were double handed and certain others like “C” were single handed [67]. In the same way, some other datasets that were used in the literature review consisted of the BVC3DSL 3-D Sign Language Dataset and other publically available datasets like HDM05, CMU, and NTU RGBD skeletal data.

## 4. Machine Learning

Machine learning is the most widely used application of AI, making the system realize the environment intelligently. ML provides the system the ability to learn and improves results through the learning process. Based upon the learning process, ML has two types, i.e., unsupervised and supervised. In supervised machine learning, the training process is involved. The machine, at first, is trained with labelled samples. Samples of input or output data are provided during the training process. Supervised machine learning is used for regression and classification.

Algorithms like Decision trees, SVM, and artificial neural networks are used to implement supervised machine learning approaches. In unsupervised machine learning, no labelled data are provided. A predefined knowledge is provided in unsupervised machine learning. The main goal of unsupervised machine learning is to find a specific pattern in input data. Unsupervised machine learning is used for clustering. Algorithms like self-organizing maps (SOM), Hidden-Markov model (HMM), and K-means clustering are used to implement an unsupervised machine learning approach. Table 2 lists and compares the advantages and disadvantages of ML approaches.

For more prediction accuracy, a more powerful class of machine learning is in practice, i.e., deep learning. Neural networks with a higher ability to derive meaningful data from complex and unreliable data are used to detect complex structures and patterns from input data that humans cannot even detect. Adaptive learning, self-organizing, and fault tolerance of a model are the current benefits of deep learning. Requiring a larger dataset, more power consumption (faster CPU/GPU) and higher computational cost (multi-layer operations) still make it effective in terms of accuracy.

Considering the sensor domain of Sign Language, primarily machine learning-based algorithms have been applied. Support Vector Machine (SVM), K-Nearest Neighbor (KNN), Decision Tree, Discriminant Analysis, and Ensemble with all of their variants were utilized among those algorithms. Some of the experimental setups also applied Artificial Neural networks (ANN) on sign language datasets. Table 3 given below lists the most frequently used machine learning algorithms described above with all of their variants. Most probably, variants of existing algorithms are formed by increasing layers dimensions or by increasing the number of neighboring nodes for feature extraction.

All algorithms mentioned in Table 3 are related to the sensor-based domain of sign language recognition systems. Sign language data originated from the smart sensor embedded data glove were analyzed through these algorithms. These algorithms were used for training recognition models. The overall dataset acquired was divided into two main streams.

1. Training Data;
2. Testing Data;

The dataset obtained was used first for training purposes. Almost 80% of the dataset was utilized for training recognition models. After that, training model accuracy was measured, i.e., how accurately datasets have trained recognition models. Most of the modern simulation tools have built-in classification learner modules that help in the training data model and display system training accuracy. After the training procedure, testing was performed; 20% of the dataset that was left after the training model was utilized for testing purposes. Dataset samples were almost different each time when any sign was captured using sensors. Therefore, repetition chances were minimal in this scenario. Testing dataset points are most likely different than training data points. This is the challenge and recognition ability of the trained model to predict new incoming data accurately. The efficiency of the system is completely dependent upon the prediction accuracy of the recognition system. The more accurately the system can classify new incoming data, the more accurate and efficient the system will be. Another challenge in the sign recognition domain is to improve system efficiency by improving the recognition and response time of sign language classifiers. Mostly in the limitation section, authors complain about the slow processing of their sign classifier. A slow response can cause the following two reasons in the sensor-based domain. One is the involvement of a bulk of sensors for capturing input sign data. Embedment of many sensor data for capturing input signs results in slow system performance. This sluggish performance is caused by a multidimensional irrelevant data burden on the recognition system. To remove this data redundancy, Principal Component Analysis (PCA) is used for dimensionality reduction. PCA suppresses multidimensional data and extracts only those data which are helpful in performing recognition smartly and efficiently. The second reason for slow data processing is the wrong choice of the training algorithm. This results in very bad system accuracy and reduces system performance as well. So before moving towards passing the sign language dataset to recognition algorithms, researchers should know the working capability, response time, calculation parameters, and data dimension of the algorithm which is going to be utilized for sign language recognition.

Vision-sensor-based sign language recognition is considered the second most important domain in posture recognition. Fundamental components of capturing user sign language postures include high-definition camera and a processing unit, normally a computer or laptop. This is not considered a good real-time recognition method as a user cannot carry a camera all the time with them to capture sign posture and a processing unit for gesture recognition. The vision-sensor-based approach is mostly used in experimental analysis-based procedures where researchers work in the lab on real-time or static sign postures.

Deep learning-based algorithms with a multi-layered structure are used in a vision-sensor-based approach for training purposes. Usually and most commonly used vision-sensor-based sign language recognition algorithms include Neural Networks, Artificial Neural Network, Convolutional Neural Network, Hidden Markov Model, Support Vector Machine, Recurrent Neural Network, Principal Component Analysis (generally utilized for dimensionality reduction), and Fourier Transform and its other versions including Scale Invariance Fourier Transform (SIFT). These algorithmic approaches are utilized to cope with emerging issues in the sign language recognition domain. Individual algorithms or a combination of two or more algorithmic techniques are used to make the system work efficiently and improve overall system accuracy.

Lastly, the hybrid approach can utilize both algorithmic domains as it is the combination of both vision-sensor-based and sensor-based approaches. A hybrid system is also used for experimental purposes because it irritates users to lift all equipment for gesture translation. The main purpose of all machine learning-based algorithmic techniques is to provide the best recognition results for the deaf-mute community.

## 5. Article Filtration and Distribution Analysis

**Figure 13:**
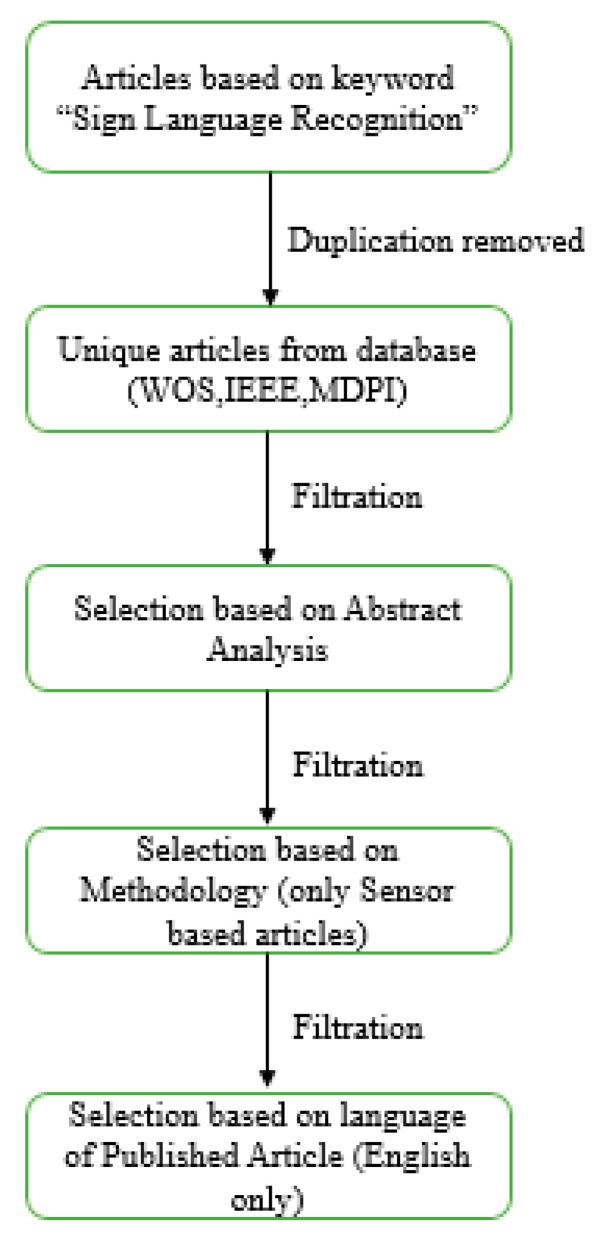
Article filtration procedure for literature review.

To perform literature review, a large variety of online databases for research articles were consulted. Among these databases, Web of Science (WOS), Science Direct, Institute of Electrical and Electronics Engineering (IEEE), and Multidisciplinary Digital Publishing Institute (MDPI) were focused on. Several journal, conference, and review articles related to the field of focus were addressed. Analyzing domain specifications, data filtration was performed, and duplicate and irrelevant research articles were removed. Further filtration was performed after studying the abstract of articles. The main goal of this study kept the focus on Sensor-based Sign language recognition using bright data gloves. The methodology section of articles resulted in further filtration as vision-sensor-based and hybrid technology-based articles were not in focus besides a few one. The last filtration procedure included articles published only in the English language. This whole method of filtration was suggested by ref. [66]. Figure 13, given below, demonstrates the filtration procedure in a straightforward way.

A complete prototype or sensor-based system embedded with the latest modules developed for the deaf-mute community was the focus of this study. So, the complete literature review was divided into categories. A major portion of this study was based on prototype development. Almost ¾th amount or 75% of reviewed articles were based on the development of such systems that could translate sign languages into texture format. These articles were based on a prototype that had been designed and implemented perfectly. Some articles focused on proposing methodologies for developing new sign language-based translation and recognition systems. Some of the highlighted articles focused on existing systems. These authors’ highlighted shortcomings and disadvantages of using such pre-developed prototypes and proposed new prototypes that were the enhanced version of existing products.

**Figure 14:**
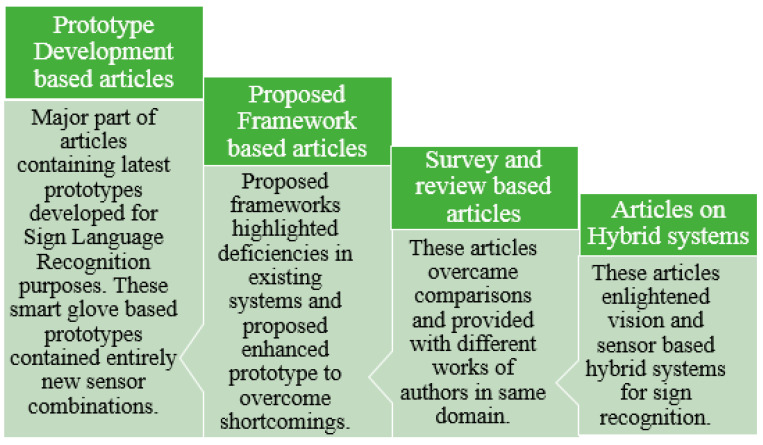
Categorical division of filtered articles used in this paper.

They were based on such a division of systems; sign language literature-based material is considered to have four main categories. These categories include development-based articles, complete design or prototype-based articles, articles based on suggested or enhanced material design based on existing prototypes, hybrid technology (sensors embedded with vision-sensor-based methodology) based articles, and literature survey or review-based articles. Figure 14 below provides an analysis of filtered articles that consisted of further classification based on metadata and approaches used in writing the article. Filtered articles were categorized according to the number of articles per domain. As most articles are based on prototype development, they lie on the left side with more area covered. Similarly, proposed frameworks, survey and review articles, and hybrid system-based articles were categorized accordingly by considering the number of articles used for this paper.

**Figure 15:**
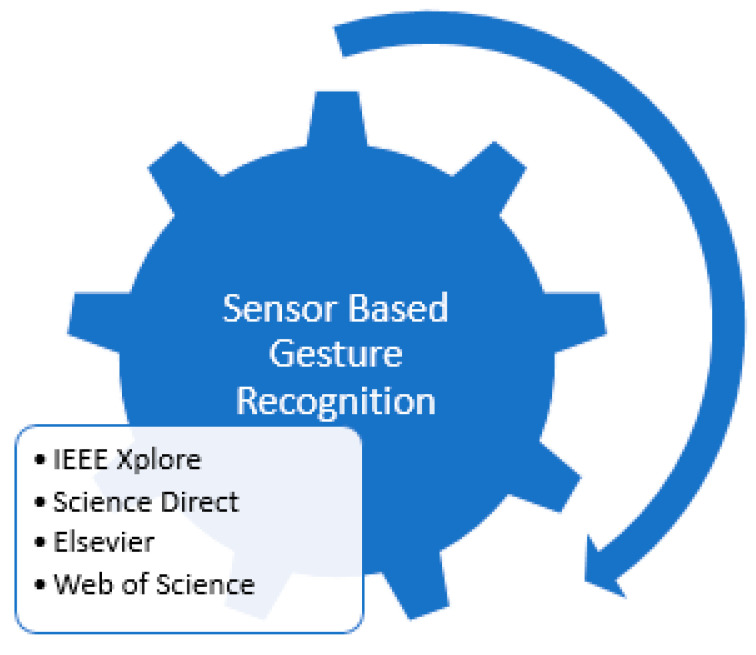
Databases used for Sign language recognition-based articles.

Results are considered the most critical parameter in analyzing the accuracy and efficiency of any system. According to the literature review, a wide range of different sensor-based combinations succeeded in achieving good results. Different databases were used for gathering data related to the sign language domain. All literature review was based on data collected from these databases. These databases included IEEE Xplore, Web of Science (WoS), Elsevier, and Science Direct. Almost half of the papers were collected from IEEE Xplore, and the remaining papers were collected from Elsevier, WoS, and Science Direct. There were many articles in the case of general search related to sign gesture recognition. That was due to the involvement of vision-sensor-based and hybrid-sensor-based sign gesture techniques. The focus of this article was based on the literature review of sensor-based sign gesture recognition. Therefore, the number of articles was reduced and limited to almost a hundred in numbers. Figure 15 nominated databases and highlighted the process of gathering data review in this article.

These articles were cited many times. Their impact factor reflected quality information and innovation presented in these articles. Table 4 lists the publisher of the article, citations of each article, and their impact factor. The impact factor of each article was extracted from the website of that specific publisher, and citations of any article were grabbed from Google Scholar.

**Figure 16:**
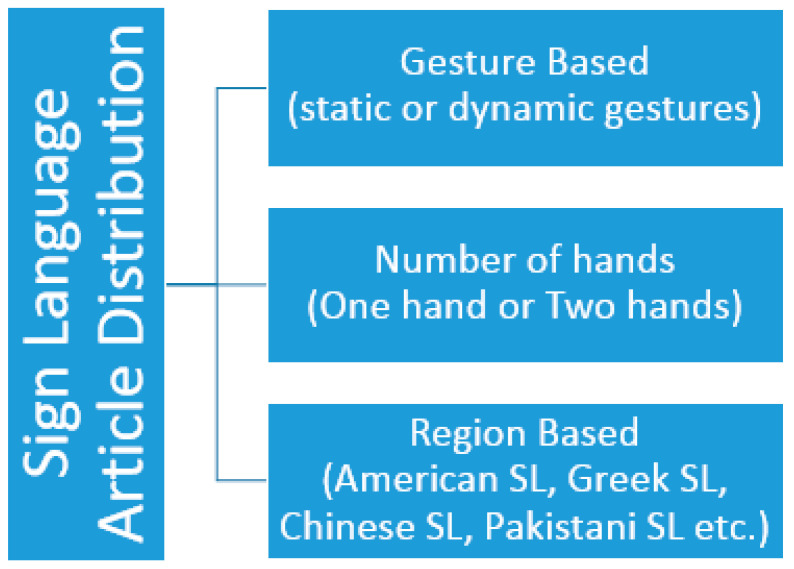
Article distribution based on region, gesture type, and number of hands used.

Articles were filtered and analyzed based on three more different parameters (shown in Figure 16) including region-based sign language recognition, type of gesture made for sign language i.e., static or dynamic gestures and analysis based on hardware module equipped on one hand or two hands.

If we considered first parameter of analysis (region-based sign language recognition) then most of articles were based on American Sign Language recognition. Almost half of the authors in this literature review had focused American Sign Language for gesture recognition. Whereas, only three or four articles were found related to Arabic Sign Language, Indian SL, Taiwan SL and Malaysian SL. Remaining regions like Pakistan, Japan, Greek, German, France, Chinese and Australia contained only one or two articles in Sign language recognition domain. Based on gesture type analysis for sign language, two main domains were located.

Sign language contained letters, words, and sentences just like any other normal language. So gestures made for letters, words or sentences divided its recognition process into two main streams. One with static gesture recognition and second with dynamic gesture recognition. Although, static gesture recognition was proved easier than dynamic gesture recognition, and therefore almost 48 researchers had worked on static gesture recognition and almost 15 researchers had worked on dynamic gesture recognition. There was another category which did not mention any of above domain and the number of articles that was found were 18. Finally, articles analysis based on number of hands used for gesture recognition were analyzed. Almost half of the researchers had used one hand for gesture recognition. Other had used two hands or did not mention the prototype in their articles.

## 6. Analysis

Sign language recognition is one of the emerging trends in today’s modern era. Much research has already been conducted, and currently, most researchers are working on this very domain. The focus of this article is to provide a brief analysis of all related work that has been done until now. For this purpose, a complete breakdown of all research activities was developed. Some authors worked on a general discussion about sign language. Most of their work was based on introduction and hypothesis to deal with sign language scenarios. There was no practical implementation of the proposed hypothesis; therefore, these authors lie in the general article category. A group of authors worked on developing systems that we are able to recognize sign gestures. This group of authors is categorized in the developer domain. A good combination of sensors was used to develop a Sign language recognition system. Most of the authors used sensor-based gloves to recognize sign gestures. Another group of authors worked on existing sensor-based models and improved the accuracy and efficiency of the system. Their focus was to use a good combination of Machine Learning and Neural Network-based algorithms for accuracy achievement. Considering the author’s intensions, Machine Learning based algorithms were used by authors working on sensor-based models like sensory gloves. The authors used Neural Network-based models working on vision-sensor-based models for sign gesture recognition.

Considering literature work, some trends were also kept under consideration. Most of the authors in the sign language domain preferred to develop their own sensor-based models. The focus of authors working in this trend was to develop their own cheap and efficient model that could detect and recognize gestures easily. These models were not made for commercial use. Authors obtained another trend to develop commercial gloves. These gloves contained a maximum number of sensors e.g., 18–20 sensors, to detect sign gestures. Cost and efficiency were the main problem in commercial gloves. Analyzing these research articles, advantages and disadvantages of vision-sensor-based, sensor-based and hybrid-based recognition models were listed. Additionally, the last trend of focus in sign language articles, including this article, was the group of those authors who worked on surveys and reviewed articles on sign recognition. These authors provided a deep understanding of research work done previously, provided detailed knowledge of hardware modules, sensor performances, efficiency analysis, and accuracy comparisons. The advantage of review and survey articles over general and development research articles was the filtered knowledge of consideration in one article. Survey-based research articles proved to be a good help for learners and newcomers in that specific topic. Survey and review articles also provided researchers with upcoming challenges, trends, motivations, and future recommendations. A detailed comparative study help use determines uses, limitations, benefits, and advancement in the sign language domain.

Based on results analysis of all literature reviews, a detailed list of accurate achievements is displayed in Table 5. It contains information about algorithms, or a combination of algorithms used collectively to gain maximum accuracy for sign language recognition techniques. Algorithmic combinations are completely case-dependent. To achieve maximum accuracy or enhanced system efficiency, authors use combinations of algorithms. The table below lists all types of accuracy results obtained in vision-sensor based, combination of different sensor-based, and hybrid-sensor-based posture recognition techniques. Most accuracy results are displayed for sensor-based recognition algorithms. Furthermore, real-time gestures and static gestures are also highlighted with respect to their results in the form of accuracy, efficiency, or outcome in Table 5.

## 7. Motivations

**Figure 17:**
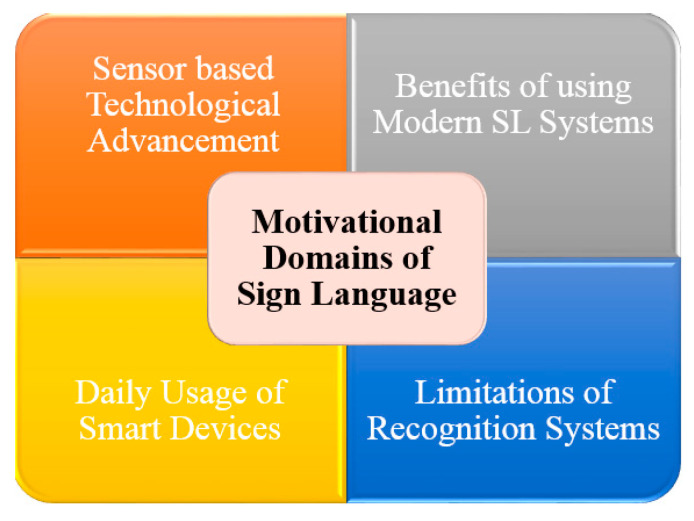
Motivational domains of sign Language.

Motivation is considered the group of multiple parameters that contributed to the continuing sign language domain using different algorithmic or sensor-based designs. Reason can also be related to accuracy and efficiency improvements. Whenever two people communicate with each other, signs and body language in sign gestures help a lot. Two ordinary people use speech and body gestures to share, but deaf or mute individuals ultimately use handmade signs or gestures to communicate with one another. Therefore, sign language is a suitable mode of motivation for researchers. Parameters that affect motivation directly or indirectly are shown in Figure 17 below. These motivational parameters include advancement or improvement in existing sign language modules, overcoming the limitations highlighted in gesture recognition, benefits, and uses of sign gesture recognition modules.

### 7.1. Technological Advancement

Technological advancement is one of the core motivations for Sign language recognition Systems developers. Despite ordinary people, defective individuals use sensor-based wearable to communicate with others. So, today’s advanced world is leaning towards more and more smart sensors day by day, thus creating a good gap for developers to enhance their recognition models. Initially designed recognition models produced less accuracy and efficiency in recognizing static or dynamic gestures. This communication gap can only be minimized by using more efficient and accurate sensors that could easily identify and recognize signs made by a mute person. Embedment of more advanced and precise sensors in existing systems enhanced system recognition ability. In the beginning, only wired high-power and slow operating sensor-based systems were developed. Then, sensor automation moved the entire wired model with wireless modules. Most recent sign language systems are completely based on wireless systems. These systems capture data from hand devices, a process that collects and transmits data towards the receiver end for gesture recognition. Previously used models had computer monitor-based display units with a populated wired environment. However, this gap was also recovered with modern LCDs and display units. Most recent studies have worked on power consumption analysis. Microchips and nanochips are now being designed to cope with energy management. This is one of the developers’ most significant challenges and core motivation to design models that utilize low power. Early work had an analog-based front end with a very complex interface. This system was difficult to understand for new users. People were made comfortable with the wearable device after proper training of developed systems. However, in today’s modern models, touch screen-based graphical user interface (GUI) with proper directions and instructions are used. This modern input device replaced old button-based models. Accuracy improvement, bend detection, hand movement, rotation measurement, hand orientation-based degree of freedom (DoF), and distance measurement improvements are good pieces of motivation for upcoming developers. This smart, fast, efficient, and low power consumption-based model helped deaf-mute individuals to communicate with others easily. Thus, this is also a big cause of motivation for developers to renovate existing models with newly equipped smart sensors, which may result in producing the best results. Systems developed approximately close to ideal systems will give a good living environment for deaf and mute people. This technology-based world will help them improve their living style and become confident in communication with other people.

### 7.2. Daily Usage

Daily usage of the sensor-based smart device covers a large domain, including an as educational tools for deaf and mute people. Despite developed countries, there are several other countries where deaf and mute people are completely unable to acquire some sort of education. Thus, lake of education also creates an increased ratio of unemployment. Therefore, this is a need for time to build a cost-effective, smart, feasible, and accurate system for those individuals who are unable to speak or hear. Using these smart sensor-based systems would become a very easy and comfortable way of learning through any source. Many developed countries are already using online learning tools, mobile applications, gesture-based animations, and video clips that help them completely correct and understand sign gestures. Successful implementation of a sign language recognition environment will remove hindrance of communication between normal and deaf-mute people. It will also act as a self-learning tool for beginners to help them train. Instructors and sign language teachers can use this sensor device for educational purposes. Affected individuals can learn sign language by observing simulators performing hand, wrist, and arm movements for sign language. Gesture recognition as an educational tool is a big motivation as well as a challenge for researchers working on or willing to work on this domain.

### 7.3. Benefits

The benefits of using a glove-based system are its ease, comfort, lightweight nature, and good man-machine interaction (MMI). Hand movements are considered the fundamental entities to perform any task or operation. Research has shown that a large number of articles have been published on human body movement, especially hand, wrist, and arm movements. Mostly sensor-based models considering hand motion are closely related to biomedical science or human engineering-based applications. Thus, the man-machine interface involving biomedical science and human engineering has been a hot and emerging domain for the last few decades. To capture hand and wrist movements, sensor-based smart gloves are always important. Considering the evaluation of technology, it is evident that sensor advancement, efficient materials, updated computing scenarios, and modern algorithmic techniques will make recognition systems more accurate and powerful. This lightweight, easy to use, efficient, and user-friendly smart equipment will be helpful in many daily life applications. A smart sensor-based module can act as an assistant for deaf-mute individuals. Other advantages of using smart data gloves are their ability to move easily with the user. Today’s technology is moving towards nano- and microchips, which have made data gloves completely independent from computer connections. Integration of wireless modules in smart systems has made the mobility of data gloves more precise and accurate. Users can move with easiness and comfort after wearing any sensor-based glove. Technological advancements of the modern era have made Man-Machine Interface (MMI) as need of time. Gesture and movement capturing environments mostly operate under the MMI domain. The main purpose of MMI is to capture and analyze signals generated after performing any operation activity like hand, wrist, or arm movement. Captured signals are converted for performance, behavior, and physiological interpretation of performed motion using active computer systems. Motion recognition or gesture recognition-based systems working on vision-sensor, hybrid, or sound-based techniques have contributed a little bit in traditional capture and recognition prototypes. The sign language gesture recognition prototype is also a man-machine interface due to its core connection of deaf-mute individual interaction with the sensor-based environment for communication purposes. A wide variety of gesture involvement in daily usage activities has made the sign language domain the most emerging field of today’s era [82]. Gesture recognition-based applications like remote controls, immersive gaming, assistive robots, virtual objects, medical health care, substitutional computer interfaces, etc., have made this field a most challenging and advanced area for researchers.

### 7.4. Limitations

Limitations in existing systems are challenges for upcoming new researchers. Nothing is made perfect; instead, perfection comes with experiment. Considering the sign language recognition environment, only two basic techniques come into mind: sensor-based and vision-sensor-based. The sensor-based smart glove contains a list of sensors to capture, analyze, recognize, and signal. However, in a vision-based approach, cameras are needed to capture sign gestures made by deaf-mute people. A lot of researchers have worked on vision-sensor-based techniques using different types of cameras like Kinect, leap motion, or sometimes a color-based glove for sign recognition. The only advantage of using a vision-sensor-based approach is the relief from warning a glove full of sensors. Considering hand orientation for sign language, the vision-sensor-based approach also captures facial expressions, object orientation, object colors, and depth knowledge. There are a lot of challenges in the vision-sensor-based approach as well. Very complex computation is required to capture gesture information from the scene. Mostly neural networks are used to cope with these complex computations. Object extraction from the background is another challenge as complex and rough backgrounds create a lot of complications in required object extraction. Therefore, it becomes difficult to discern the signs made from irregular backgrounds. Lightening conditions, luminance, and brightness also affect system accuracy and efficiency. All processing is performed via a computer with good specifications and processing ability.

**Figure 18:**
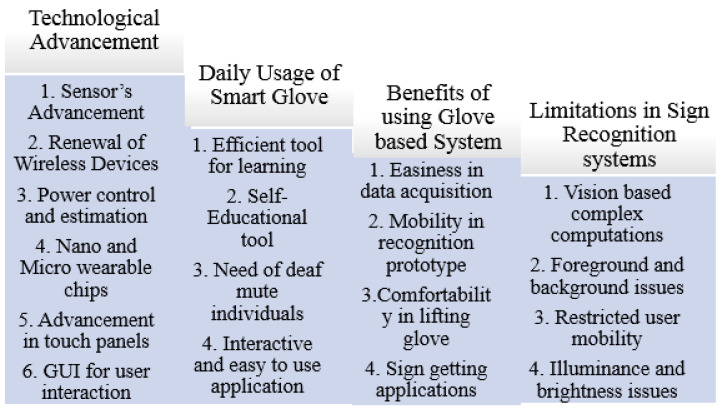
Overview of key points regarding motivational domains of sign language.

Most importantly, individuals with speech and hearing disabilities will always require a camera to lift with them for gesture capturing purposes and also a display unit to display recognized results. This sort of experimental arrangement will completely affect the daily life activities of the deaf-mute community. A quick overview of all detailed discussions regarding motivational domains of the sign language recognition system is described in Figure 18. The flow-based list in Figure 18 summarizes the whole discussion in one format for a quick understanding of motivational domains.

## 8. Challenges

**Figure 19:**
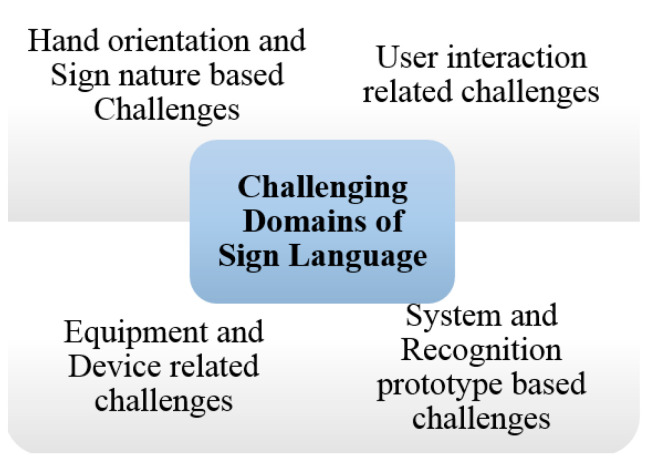
Challenging domains of sign language.

Challenges in the sign language recognition system played a vital role in increasing system accuracy. Any challenge faced by researchers received the universal attention of researchers working in the same domain. Successful implementation of provided challenge help in improved system accuracy and may also highlight some other sort of challenges. Considering the literature review, a deep analysis of challenges regarding the sign language recognition system is obtained. Issues and difficulties faced by developers are analyzed thoroughly. This section contains challenges regarding sensor-based, vision-sensor-based, and hybrid systems equipped for SL recognition. Based on the literature review, the challenge domain was categorized into four main streams: nature and orientation of signs, system characteristics of sign language recognizers, and challenges related to the user, sensors, and device characteristics. An overview of challenging domains for sign language recognition systems is given below in Figure 19.

### 8.1. Sign Nature

Sign nature and hand orientation are primary considerations in making sign language gestures. Considering any sign language based on any region, their domain is divided into two basic streams: static sign and dynamic sign. Most of the literature review is composed of the development and analysis of static signs, and very few researchers and developers have worked on dynamic gestures for any sign language. This is because static sign gestures are comparatively easier than dynamic ones, and fixed sign accuracy due to better classification is always greater than dynamic sign gestures. Insufficient training and no track of movement history cause low accuracy and a higher error rate of dynamic sign gestures. The similarity of some gestures is another challenge of the sign language domain. Considering American Sign Language alphanumeric gestures, some alphabets like M, N, S, and T have the same posture.

Initially, all these mentioned signs are made with a closed fist, making a very low change in orientation. Alphabet U and V are also categorized into this very domain. Here finger and palm orientation are almost similar. These similar gestures become the cause of misclassification and lead towards low system accuracy. Another challenge in sign recognition is related to the continuous sign domain. Continuous signs are performed by individuals on a specific delay and transition time. So, these delays are calculated before concatenating the whole gesture. Mostly words and phrases are gestured using continuous signs. Words concatenation as speech without any discontinuity is the main issue to be faced in dynamic sign gesture recognition systems. Similarly, the transition from one gesture to another gesture consumes some time. This transition delay is also a big challenge as movement frames are created within gesture sequences that are meaningless and capture space. This is also considered a classification issue, and the frames lost are normally termed as “Movement Epenthesis”. Region-based recognition systems are not identical, and every region has its own gestures corresponding to sign language. Therefore researchers have not listed this issue in global challenges. In a nutshell, it can be stated that there is no universally ideal system for sign language that can cope with sign gestures of all regions. Despite standard static alphanumeric American Sign Language systems, all universally dynamic sign language systems are different due to different languages around the globe. Therefore, there can never be a standard sign language system for the whole world.

### 8.2. User Interactions

User interaction-related challenges are faced while performing gestures. The tradeoff between a beginner and an expert is kept under consideration. A beginner can never perform sign postures perfectly as compared with an expert one. If any beginner can make correct orientation for sign postures, it would be somehow difficult for the listeners to understand that sign postures perfectly. Using a sign language system as a learning tool, hand orientation, finger movement, wrist, and joint movements are kept under deep consideration. Performing as an experiment tool, one signer is asked to make a single gesture multiple times because the same individual cannot complete the same posture similarly in multiple attempts. This is known as signer dependency because a single individual cannot perform the same gesture and place all wrists, joints, and fingers in the same place all the time. Therefore, the value varies, and a range of different values is taken corresponding to a single posture during the training phase. Among user and gestures differences, another problem is the size and shape of the user’s hands. Typically, data gloves are made of a specific size. Leather-made sensor embedded data gloves are used universally for static or dynamic gesture capturing. Tall or short, slim, or fat, and small or big hands will also create problems in data acquisition. These user interaction-based issues affect the training process of acquired data and reduce overall system efficiency by interpreting more minor system accuracy.

### 8.3. Device Infrastructure

Device infrastructure and prototype-related challenges are the core factors in the human engineering development model. Among hardware challenges, price and power are the most crucial ones. A large number of companies have already developed smart sensor-based gloves that can capture and display signs made by deaf-mute individuals. However, the price of the international prototypes ranges from USD $1000 to $20,000. This range of price is beyond the range of any common community to buy and use it. The facts show that the deaf-mute individuals mostly belong to middle-standard families among the defected community. So, this looks like a dream for these people to use these smart devices for communication purposes.

Portability is another challenge in sign language recognition. Most of the postures are very complex and need some computational time for gesture recognition. Multiple gestures are combined collectively to make words or sentences. So, in that case, computational work exceeds even more, and it could not be processed without the help of a computer. Practically, it is impossible to lift computers everywhere for SLR. This phenomenon makes the overall prototype limited with respect to portability. Considering only American Sign Language ASL alphabets and numbers, most of the alphabets and numbers are postured with palm and finger orientations. Combining alphabets as words or sentences is tricky to capture with only finger and palm movement. For this purpose, the movement of hands, wrists, arms, and elbows is also involved. Sometimes the action of lips is also captured for recognizing sign gestures.

Sensor-based gloves are capable of capturing only finger and palm data. The data related to the elbow, wrist, and arm are not captured, and hence are a challenge for the researchers. This missing data also create problems in recognizing motion-related data. For example, the alphabet ‘j’ and ‘z’ are related to the motion of the arm; wearing a data glove will not be able to capture data of these two alphabets as arm, and other body parts related data are not captured with the sensor-based data glove. Talking about prototype performance seemed to be another emerging challenge as most sensor-based smart prototypes provide a detailed analysis of sensor efficiency and accuracy. But overall recognition accuracy and prototype efficiency are not described. There is no common standard of utilizing the accuracy of data obtained from gestures. Systems are lack methodologies that would help in determining standardized entities for performance and accuracy analysis of the system. Contributing to the achievement of true data and pure systems efficiency will help society and researchers.

Another challenge in the SLR domain is to achieve the best and desired results with minimum cost. For the sake of minimizing system cost, hardware equipment involved in designing prototypes loses its quality. Low-quality sensors are used for minimizing overall system cost. These low-quality sensors produce faulty data as noise and divert the actual resulting values. In this way, overall system accuracy is decreased. So, noise removal is another big challenge in acquiring authentic and accurate data. Universally, the physical appearance and body structure of humans vary. Some of them are fat, tall, thin, or small. Based on physical appearance, hand size, fingers appearance, and thickness or thinness of hand also vary. Size fluctuation affects recognition performance directly as sensors attached to the finger overlap, and the actual reading of made gestures becomes faulty. Therefore, sign data gloves must be calibrated for different age and hand size users. User training and testing are also performed to cope with this challenge. During the training session, the user is directed to complete specific sign postures to calibrate equipment utilized for recognition purposes.

Among described challenges, the real and actual challenge in sign language recognition is to utilize minimum sensors that can perform with maximum accuracy. However, a very low number of sensors will lose posture information that posing hand will make. In this way, the system accuracy will be affected and will lead to low system accuracy. Another consideration in prototype designing is to keep track of sensors used. The prototype must not include as many sensors that can put the burden on the processor and reduce its task execution capability. Sensor selection is another challenging task in the posture recognition domain. There are a majority of sensors in the market that have been used for hand posture recognition. Not all the sensors are good in their field of view. Every sensor has its own good and bad impact on overall system accuracy. In the wide range of sensors, every sensor has its own way of measuring finger bend. All these ways of capturing finger bends are acceptable but not efficient in all domains. So, the challenge is to find the sensor with the best bending results in the recognition system. Another important challenge is placing desired sensor components on data gloves as sign language is completely based on the movement of the hand, wrist, palm, elbow, and arm. Therefore, the correct placement of bend detection-based and finger orientation detection-based sensors is critical. Correct placement of sensors with the best working ability will enhance system efficiency and produce maximum recognition system accuracy.

### 8.4. Accuracy

Accuracy-based challenges are prevalent in any research domain. Initially, sign language recognition systems, either static or dynamic, faced terrible accuracy. Like other research domains, this was the main challenge to overcome. For this purpose, instead of covering complete body movement including arm, wrist, elbow, and fingers at once, a partial system was introduced to capture posture movements in chunks. Moreover, the existing glove-based model was renovated to increase overall system accuracy. Sign language was analyzed from the very beginning in different formats. There are a lot of techniques that focus on static sign language recognition but finding best portable solution for a real-time gesture recognition system is still a challenge.

Real-time recognition systems must be able to be more precise, fast, efficient, and accurate to cope with the speed and processing of real-time gesture translation. Despite static postures, dynamic or real-time sign posture must deal with a randomized hand degree of freedom. Exceptional and unwanted data make the process challenging to map incoming input data. Although modern processors are very fast, modeling real-time input data at a time makes it difficult for them to process. Dataset generation or acquisition is another major challenge of research domains. This is the most important consideration and yet is ignored. The availability of an accurate dataset is complicated in the sign recognition domain. The number of available datasets is deficient, and the available datasets are not up to the mark. An accurate dataset is available to the researchers that saves dataset generation and accuracy precision time. However, if the previously used dataset is utilized, it may result in poor system accuracy.

**Figure 20:**
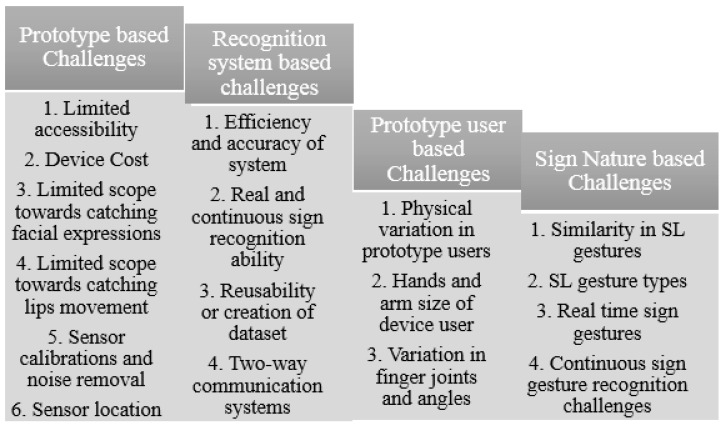
Key points of challenging domains of sign language models.

Another major challenge in sign language recognition is to design two-way communication systems. Most of the sign language domain designs are educational models for teaching sign languages. The international market is full of such prototypes which help in teaching sign languages. However, two-way communication-based real-time sign language translators are very few in number. Prototypes designed as educational tools are not suitable to cope with two-way communication. However, these prototypes, including all types of sensors, are boosting processing speed and system accuracy, which is good work done by researchers. Very few sign recognition models have not embedded flex, pressure, and contact sensors. Instead, they worked differently and formed simulation-based models, which produced good efficiency and accuracy. All discussed challenges are listed in Figure 20.

## 9. Recommendations

**Figure 21:**
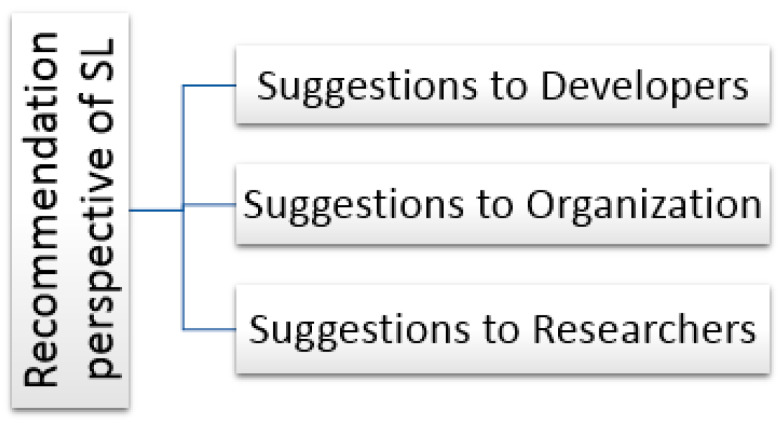
Recommendation perspective of sign language.

Recommendations play an essential role in increasing system efficiency and demand worldwide. Recommendations are the suggestions that help in improving system design, accuracy, prototype physical appearance, and graphical user interface. All these improvements are based on two parameters: one is the user or the public feedback, and the other is personal or organizational provided analysis. Considering sign language recognition systems, there are a lot of people with hearing and speech disabilities. This creates a communication gap between the society and the affected community. Thus, this is a challenge to the developed society as well. To overcome this hindrance, a lot of systems have been developed. These systems are used globally. Based on user and organizational reviews, recommendations and suggestions are categorized into three different domains, as shown in Figure 21 below. These three domains are the recommendations/offers to the researchers, developers, and public or private sector organizations. To cope with emerging challenges and user expectations, it has become necessary for every developer of the sign language domain to keep in mind that recommendations or suggestions for gaining high accuracy and efficiency.

### 9.1. Developers

Developers are the main contributors in designing sign recognition prototypes. There are a large number of different parameters which affect system performance directly or indirectly. All parameters are under the development wing, which developers of the system handle. Therefore, suggestions to the developers are on top of the recommendation section. Cost is one of the core challenges to the developers. According to a literature survey, most of the world population affected and unable to speak or hear belong to the middle class or low class. So, cost control is highly recommended to the developers. Sign recognition devices must be cheap, reliable, and efficient to perform accurate sign recognition. Another important suggestion is about system reliability and accuracy. A core feature of the recognition device is to capture and recognize sign gestures perfectly and accurately. The system with more accuracy will help people understand gesture-based language and recover the communication gap between society and the affected individuals. So, a sign language recognition system with the best reliability and highest accuracy is the most demanding parameter. If the overall system performance is enhanced, the error ratio is minimized automatically.

Suitable quality sensors with the highest accuracy can increase system reliability, mainly used in sign language recognition prototypes, flex sensors, three-axis accelerometers, and gyro sensors. A system with these best-quality sensors will produce maximum accuracy results. A high-quality output module is also recommended for a good user experience. In the sigh recognition prototype, a speaker and some graphical user interface GUI are involved. The purpose of the speaker is to transmit recognized posture into speech format so the other person can understand the meaning. For this purpose, the speaker must be loud and clear.

Regarding device usability, the recognition device interface must be user-friendly. The signer must not feel any sort of difficulty in using a prototype. They must also be capable of verifying gestures made by the signer. This will help in the training of newly interacted deaf-mute individuals with the recognition device. GUIs must also be of good quality. Alphabet, words or phrases obtained after making gestures should be displayed perfectly and timely. A good GUI will affect output performance and also increase user attention. 3D sign animation is now becoming the focus of market attention. For this purpose, a proper market analysis-based animation module is required to fulfill user requirements without adding much in cost. Introducing wireless display modules in the sign recognition domain has opened up new display options for developers. The most commonly used wireless module is the smartphone. So, considering smartphones as a wireless output display unit, a beautiful graphical interface, easiness, friendly intractability, and intelligent applications are required. Successful implementation of these features in the sign recognition domain will effectively boost system performance. The future of the sign language domain resides in real-time gesture recognition. Most of the authors have already worked on real-time recognition. It is also recommended that the developers design such real-time applicable systems that can interact with other people without any deal. A real-time system must assist the user and provide urgent feedback to another person. One hand can easily make most sign language postures, including alphabets and numbers. However, regional-based sign language postures involve two hands. This is a challenge and recommendation to the researchers to expand the sign language domain by introducing a perfectly efficient two-hand data glove for sign recognition.

Considering material and sensor attachment on data glove, it is also recommended to use excellent, comfortable, and easily stretchable material for making data gloves. As deaf-mute individuals have to use data gloves almost all the time, the material must be comfortable to use. Data gloves contain sensors on each finger and on palm, so this glove should be waterproof to save any sort of short circuit due to rain, water, and sweating. Glove size is another issue to be addressed. People with different hand and finger sizes use gloves. Therefore it is recommended to use almost all sizes of gloves and adjust sensor size accordingly. Material and data glove calibration must be performed on real-time gestures as there are a lot of variations on values in real-time and virtually simulation time. Real-time sensors continuously emit values and also get affected by the environment. Therefore it is recommended to calibrate the data glove according to all angles and sensor values. Smart data glove for sign translation is used everywhere by affected individuals, so their design must be the latest and up to date. People working in public and commercial places should not feel embarrassed while wearing data gloves. So, its material, design, style, and technological involvement must be up to the mark. Lastly, it is recommended to develop portable recognition devices to assist deaf-mute individuals to cope with their daily routine activities without getting connected with any physically available computer device.

### 9.2. Organization

Organization is the critical factor in developing prototypes used for sign language recognition. Sign language recognition is one of the most emerging domains. A lot of researchers are already working in this sector. Most of the research organizations are working on real-time applications related to sign language recognition. These real-time applications are implementable on very perspectives directly or indirectly associated with sign recognition. Organizational impact on different sectors will collectively benefit the deaf-mute community and the common public. Targeting public places like bus stations, railway stations, hotels and restaurants, public offices and banks, airports, and hospitals where individual communication is at its peak, organizations could help by providing sign language recognition devices for enabling feasible gesture-based communication. Organizations related to the medical and surgery department can also use these sign recognition devices to facilitate their staff. This could be helpful during surgical operations and staff communication by improving system performance and accuracy. Automation and industry sectors can also utilize sign recognition devices as operational tools. This could also help in machine and equipment maintenance and robotic operability. Organizations and public sectors working on virtual reality-based interactive environments can utilize sign recognition-based data gloves to interact with the environment remotely. Virtual reality-based data gloves can control computer games, home appliances, and act as a mouse for laptops and personal computers. Virtual keyboards and musical instruments can be operated using glove-based innovative technology. Most importantly, educational sectors and training institutes can use sign recognition devices as a learning tool for deaf-mute communities, especially children. They can help them learn words, alphabets, numbers, prepositions, and sentences. Smart sensor-based data gloves can help them communicate with the public and remove social distances effectively.

### 9.3. Researchers

Researchers are the main working body of any development team. There are a lot of considerations that must be kept in the minds of researchers, so it may help to develop approximately perfect systems. Among these considerations, researchers’ first and foremost recommendation is totally about database generation. A vast collection of all dataset variants, including numbers, alphabets, and words, is required for good research. The extensive dataset will provide better training results than the lower dataset from a machine learning perspective. It is also essential to choose high-efficiency data for dataset perspective. So, it is somehow a big challenge and future recommendation to researchers to work on high-accuracy and high-efficiency datasets. Dataset availability is another consideration in gesture recognition systems.

People working in the sign language recognition domain have access to a minimal dataset. Most of the time, researchers in sensor-based specific environments tend to have their dataset. However, researchers working on vision-sensor-based fields mostly use a predefined dataset provided publicly. However, due to the limited dataset, the accuracy of the dataset is also a big challenge. So, it is recommended to create efficient datasets and give the public access to other researchers. Public datasets available are not adequately classified.

Datasets are available in only digit or alphabet format. Very few researchers have worked on words and phrases posture recognition. So, it is recommended to enhance the sign language dataset concerning numbers, alphabets, words, and sentences. Like spoken language, sign language also has a series of rules and gesture-based procedures to form any phrase or sentence. In the formation of a sign language recognition system, these identical and isolated rules must be followed as they will help in developing an efficient and reliable translation system. Another important recommendation is to keep good consideration of different regional-based sign language systems. Every region’s common communication-based sign language is different from that of other areas. So, it is a recommendation and a challenge to develop region-based isolated sign language translation systems. In this perspective, it was recommended by a researcher to develop a standard universal translation system with specific rules that could be accessible and usable by any deaf-mute individual around the world. It is also recommended to introduce hybrid sign language recognition and translation systems. Almost all researchers had worked on either sensor-based or vision-sensor-based SLR approaches. These vision-sensor-based and sensor-based approaches have their own merits and demerits. However, the combination of both sensor-based and vision-sensor-based techniques will overcome the demerits of both technologies.

A hybrid approach implemented on two hands to identify facial expressions will provide better results—sensor fusion-based prototypes help in capturing all movements performed for sign formation. A flex sensor on each finger and a gyroscope with an accelerometer sensor will provide bending and axis orientation of hand movements. To reduce the ambiguity of differentiating similar postures and misspelling, a filtration process with the embedment of pressure sensor attached on the middle finger is utilized. Moreover, facial expressions and body motion are also captured for proper language translations.

To capture complete posture information made for sign language, elbow and shoulder joints are equipped with sensors to acquire movement data. Minor information regarding capturing facial expressions, data fusion between two hand-based sensors gloves, and head and body movement for posture formation are also captured. The role of the threshold value is very considerable in a vision-sensor-based recognition system. Threshold points act as a filter for input data. It helps in capturing desired data and discarding false input data. For multiple feature environments, the double threshold can also be applied to capture real sign data and discard unwanted inaccurate input data.

Most of the communication errors are removed through this method. Therefore, it is recommended to keep the threshold under good consideration. Using a reasonable and feasible number of sensors impacts system accuracy. Using a lot of sensors results in increased system complexity. A lot of combinations are used to capture hand motion. For this purpose, normally, flex, contact, pressure, accelerometer, and gyroscope-based sensors are used. This combination of sensors has the ability to capture very minute changes in hand. Therefore, it is recommended to utilize these sensors to find good recognition results with improved accuracy and increased efficiency. More sensors are required to capture real-time sign recognition systems with minor details, including head movements, facial expressions, body postures, and hand motions. The involvement of more sensors in real-time recognition systems helps in increased accuracy of enhanced efficiency systems. It is also recommended to merge all sensor data into one format for easy processing of recognition algorithms.

**Figure 22:**
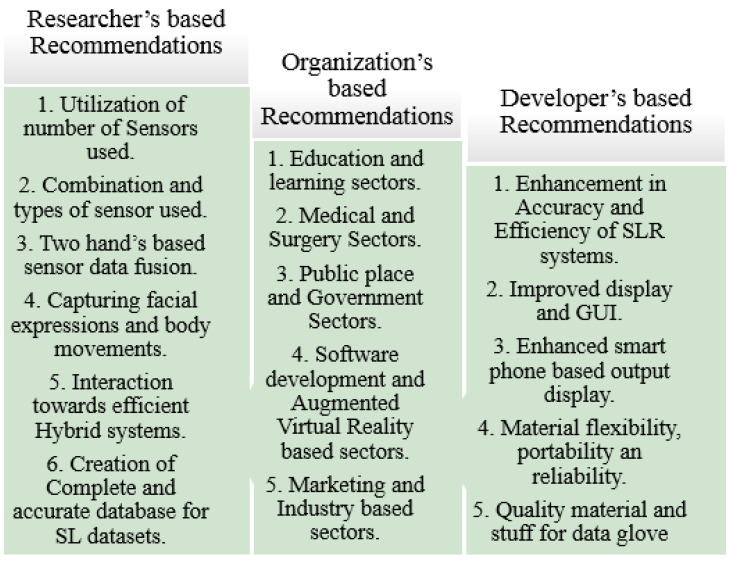
Overview of recommendation domains of the sign language model.

It is also recommended to use minimum sensors that can cope with all system functionalities. This will help reduce system complexity and hardware fusion issues. Figure 22 provides an overview of Sign language recognition’s recommendation domain. These recommendations to developers, organizations, and researchers will play a vital role in improving overall system accuracy. This will also help increase user demand by interacting and fulfilling public needs.

## 10. Conclusions

Developing an automatic machine-based SL translation system that transforms SL into speech and text or vice versa is particularly helpful in improving intercommunication. Progress in pattern recognition promises automated translation systems, but many complex problems need to be solved before they become a reality. Several aspects of SLR technology, particularly SLR that uses a glove sensor approach, have been previously explored and investigated by researchers. In this paper, an in-depth comparative analysis of different sensors in addressing and describing the challenges, benefits, and recommendations related to SLR was presented. The paper discussed the literature work of other researchers mainly targeting the available glove types, the sensors used for capturing data, the techniques adopted for recognition purposes, the identification of the dataset in each article, and the specification of the processing unit and output devices of the recognition systems. The comparative analysis would be helpful to explore and develop a translation system capable of interpreting different sign languages. Finally, datasets generated form these sensors can be used for tasks of classifications and segmentations to assist in continuous gestures recognition.

## Figures and Tables

**Table 1:** Basic elements of sign language recognition.

Entity	Attributes
Face Expression	Happy, angry, sad, excited, and wondering face with or without movements of the lips and head.
Orientation of hands	Up, down, inward, and flipped palm orientation.
Hand movement type	Forward, backward, left, and right hand movement.
Configuration of hands	Finger movement and bending with or without palm bending.
Hand articulation points	Finger, wrist, elbow, and shoulder joints.

**Table 2:** Machine learning approaches: a comparison.

ML Approach	Advantages	Disadvantages
Supervised machine Learning	Defined data classes with labelled data, making it easier to learn and classify with more accurate results	Labelling data by humans which is not appropriate for the system to operate automatically. Computationally expensive. Required dataset for training and testing.
Unsupervised machine Learning	No labelling of data. No training data are required. Accurate results for new or unseen objects	Can produce less accurate results due to no labelled data. Do not provide details.
Deep Learning	Feature engineering work is reduced which is time taking and extracting information more accurately which are even hidden from the human eye.	Requires large dataset to train and computationally much more expensive.

**Table 3:** Machine learning algorithms with variants.

Sr. No	Algorithm	Algorithmic Variants
1	Decision Tree	Simple Tree Medium Tree Complex Tree
2	Discriminant Analysis	Linear Discriminant Quadratic Discriminant
3	Support Vector Machine (SVM)	Linear SVM Quadratic SVM Cubic SVM Fine Gaussian SVM Medium Gaussian SVM Coarse Gaussian SVM
4	K-Nearest Neighbors (KNN)	Fine KNN Medium KNN Coarse KNN Cosine KNN Cubic KNN Weighted KNN
5	Ensemble	Ensemble Boosted Trees Ensemble Bagged Trees Ensemble Subspace Discriminant Ensemble Subspace KNN Ensemble RUSBoosted Trees

**Table 4:** Impact factor and citation analysis of reviewed articles.

Article Reference	Article Publisher	Impact Factor	Citations
[71]	IEEE Access		45
[68]	IEEE		7
[65]	IEEE		3
[43]	IEEE Access		2
[67]	IEEE Access		15
[34]	IEEE Access		18
[74]	IEEE Access		21
[60]	IEEE		
[37]	IEEE Access		2
[107]	IEEE		9
[61]	IEEE Access		34
[45]	IEEE Access		5
[46]	IEEE		8
[66]	IEEE		19
[64]	IEEE		37
[42]	IEEE		44
[75]	IEEE		12
[73]	IEEE		3
[41]	Advanced Robotics Journal		1
[38]	IEEE Transaction		65
[39]	National Library of Medicine		
[35]	Journal of Informatics		22
[107]	IEEE		9
[37]	IEEE Access		478
[35]	Informatics	2.90	281
[26]	Procedia Engineering	3.78	112
[58]	International Midwest Symposium on Circuits and Systems		83
[65]	IEEE Access		60
[56]	International Conference on Engineering and Technology		51
[32]	Journal of King Saud University of Computing and Information Sciences	5.62	43
[20]	Texas Instruments India Educators’ Conference		56
[28]	Computer Science applications	2.90	33
[21]	IEEE		30
[29]	Global Summit on Computer & Information Technology		30
[51]	Experimental System Applications	2.87	25
[50]	International Journal of Computing		22
[52]	International Journal of Computer Science and Engineering		21
[30]	IEEE		20
[47]	Texas Instruments India Educators’ Conference		20
[38]	IEEE Transaction		18
[44]	IEEE Access		17
[63]	Conference Proceedings Paul Cunningham and Miriam Cunningham (Eds)		17
[12]	IEEE		16
[16]	IEEE		16
[54]	International Conference on Advanced Information Networking and Applications Workshops		16
[3]	International Journal of Advanced Research in Electronics and Communication Engineering	0.77	14
[55]	Computer Vision Images		14
[23]	IJECT		13
[25]	IEEE		13
[31]	International Conference on Neural Information Processing		13
[61]	IEEE Access		13
[9]	International Journal of Engineering Sciences & Management Research		11
[45]	IEEE Access		11
[57]	Procedia Computer Sciences		11
[13]	International Symposium on Scientific Computing, Computer Arithmetic and Validated Numerics		10
[19]	International Conference on Control, Automation and Systems		10
[24]	2011 International Conference on Body Sensor Networks		10
[44]	IEEE Access		9
[11]	IEEE Transaction		9
1	International Conference on Contemporary Computing		8
[6]	Procedia Computer Science Applications	0.29	8
[8]	International Journal of Scientific & Engineering Research		8
[64]	IEEE Transactions		8
[4]	International Colloquium on Signal Processing & Its Applications		7
[5]	International Journal of Computing Applications		7
[18]	International Conference on Advances in Electronics, Computers and Communications		7
[40]	IEEE Sensors Journal		7
[7]	International Journal of Innovative Research in Computer and Communication Engineering		6
[14]	International Conference on Wearable and Implantable Body Sensor Networks		6
[15]	International Conference on Control, Decision and Information Technologies		6
[27]	International Journal of Information Technology	0.80	6
[33]	International Conference on Electrical Engineering, Computing Science and Automatic Control		6
[59]	Pattern Recognition	3.60	6
[60]	IEEE Access		6
[12]	IEEE		5
[34]	IEEE Access		5
[66]	IEEE Signal Processing Letters		5
[67]	IEEE Access		5
[22]	IEEE		4
[43]	IEEE		4
[46]	IEEE		4
[49]	IEEE		4
[36]	IEEE Sensor Journal		3
[41]	Advanced Robotics		3
[42]	Sensors Journal		2
[48]	International Conference on Electronic Devices, Systems and Applications		2
[53]	Software Computing Applications		1
[62]	IEEE Sensors Journal		1

**Table 5:** Results analysis of algorithms used for classification and recognition.

Ref	Classification and Recognition Algorithms	Results (Accuracy/Efficiency/Outcome)
[71]	3-dimensional residual ConvNet and bi-directional LSTM networks	89.8% on DEVISIGN_D dataset and 86.9% on SLR dataset
[34]	Convolutional self-organizing map	89.5% on Deep Labv3+ hand semantic segmentation
[37]	Support Vector Machine (SVM)	91.93% recognition accuracy
[61]	PCA and SVM	88.7% average accuracy by leave one out strategy
[45]	Aligned Random Sampling in Segments	Recognition accuracy of 96.7% on CSL dataset and 63.78% on IsoGD dataset
[46]	Gradient Boost Tree with Deep NN	Recognition accuracy over 98.00%
[42]	LSTM Model	89.5% on isolated sign words and 72.3% on signed sentences
[41]	LDA, KNN, and SVM	98% average accuracy on ASL
[38]	Wrist based gesture recognition system	92.66% on air gestures and 88.8% on surface gestures
[35]	Local Fusion algorithm on motion sensor	F1 score of 91%, mean accuracy of 92% and 93% gyro-to-accele ratio on LSF data
[57]	Orientation Histogram and Statistical (COHST) Features and Wavelet Features based Neural Network	Recognition rate of 98.17%
[58]	Wavelet transform and neural network	94.06% on sensitivity of gesture recognition
[51]	Hough transform and neural NN	92.3% recognition accuracy on ASL
[52]	B-Spline Approximation and support vector machines (SVM)	90% for alphabets and 92% for numbers on average 91%
[53]	Hybrid pulse-coupled neural network (PCNN), non-deterministic finite automaton (NFA) and “best-match”	96% on pose invariant restrictions
[54]	Local binary patterns (LBP) and principal component analysis (PCA) Hidden Markov Model (HMM)	93% recognition accuracy
[56]	Local Binary Patterns, Principal Component Analysis, Hidden Markov Model	99.97% signer independent recognition accuracy
[15]	Matching technique	Voice and Display based output
[16]	template matching	Computer display based output
[17]	HMM-based model	Text-to-speech based outcome
[18]	Matching technique	computer display and voice based output
[83]	HMM and Parallel HMM	99.75% recognition accuracy
[19]	Matching algorithm	92%
[25]	statistical template matching with LabVIEW Interface	95.4% as confidence intervals
[27]	Statistical Template Matching	69.1% accuracy with LMS and 85% for excluding ambiguous signs
[28]	matching template	89% in case of translating all gestures, 93.33% for numbers, 78.33% for gesture recognition and 95% overall average accuracy.
[30]	selection-elimination embedded intelligent algorithm	System efficiency was enhanced from 83.1% to 94.5%
[84]	artificial neural networks (ANN)	92% and 95% accuracy for global and local feature extraction
[31]	ANN	89% recognition accuracy for sentences and punctuation
[32]	Hand segmentation, tracking feature extraction, classification for skin blob tracking	97% recognition rate for signer independent platform
[33]	NN based cross validation method	96.1%
[89]	Modified K-Nearest Neighbor (MKNN)	98.9%
[90]	decision tree and multi stream hidden Markov	72.5%
[8]	Motion sensor-based matching system	Voice and display based output
